# Robust Arduino controlled spin coater using a novel and simple gravity chuck design

**DOI:** 10.1016/j.ohx.2023.e00422

**Published:** 2023-04-17

**Authors:** Adam Shnier, Francis Otieno, Caren Billing, Daniel Wamwangi, David G. Billing

**Affiliations:** a School of Chemistry, University of Witwatersrand, Johannesburg 2050, South Africa; b School of Physics, University of Witwatersrand, Johannesburg 2050, South Africa; c DSI-NRF Centre of Excellence in Strong Materials (CoE-SM), University of Witwatersrand, Johannesburg 2050, South Africa; d Department of Physics and Materials science, Maseno University Private Bag Maseno, Kenya

**Keywords:** Spin coater, Arduino, Gravity chuck, Thin film, Low cost

## Abstract

Spin coaters offer an invaluable method of thin film fabrication. Various implementations, both proprietary and open-source exist, offering vacuum and gravity samples chucks. These implementations vary in their reliability, ease-of-use, cost, and versatility. Here we present a novel easy-to-use open-source gravity-chuck type spin coater with minimal points of failure at a material cost of around 100 USD (1500 ZAR). The unique chuck design makes use of interchangeable brass plate sample masks, each specific to a sample size, these can be made with basic skills and common hand tools. In comparison, replacement chucks for commercial alternatives can cost as much as the entire spin coater we present. Open-source hardware such as this provides an example for individuals in the field on the design and development of hardware where reliability, cost, and flexibility are most important, as is the case for many institutions in developing countries.

## Graphical abstract

**Figure:**
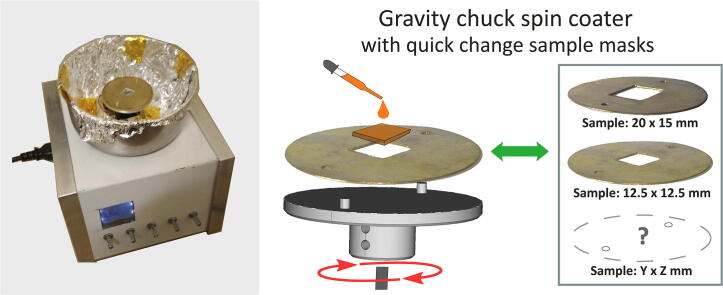

**Specifications table**

**Table:** 

**Hardware name**	*Gravity chuck spin coater*
**Subject area**	• *Engineering and material science* • *Educational tools and open source alternatives to existing infrastructure*
**Hardware type**	• *Mechanical engineering and materials science*
**Closest commercial analog**	Ossila Spin Coater [1]
**Open source license**	Software: MIT Hardware: SHL-2.1
**Cost of hardware**	$ 80 - $ 120
**Source file repository**	Mendeley Data, http://doi.org/10.17632/y84tbnfsj4

## 1 Hardware in context

Spin coating is a widely used process for the deposition of uniform thin films on flat substrates from a precursor solution. The technique is common for fabricating thin films of photovoltaic materials, small organics molecules, polymers, nanoparticles, metal oxides, etc. [2], [3], [4]. A substrate is mounted on the spin coater then a liquid precursor is applied to cover the substrate. The spinning process drains precursor solution from the substrate using centrifugal force, while viscosity and surface tension restrict this process [5]. The film thickness and quality is affected by rotational speed, atmosphere, solvent evaporation, the rheology of the precursor, and surface wetting of the substrate [2], [5].

Commercial spin coaters are most commonly available with either gravity or vacuum chucks to mount the sample. A vacuum chuck exposes a sample to a low vacuum through an opening in the sample chuck. The size and shape of the chuck varies, with the simplest implementation being a small hole in the centre of the chuck. One advantage of vacuum chucks is the ability to accommodate any sample shape and a wide range of sizes, provided the sample’s base can make a good seal against the chuck. Vacuum chucks require a vacuum pump and a *rotary union* suitable for high speeds, adding both cost and complexity. Another consideration is that the suction of a vacuum chuck may warp very thin substrates (such as 300 μm Si) [1].

Gravity chucks typically take the form of an inset shape matching a specific sample size. These may require either a different chuck entirely for each sample size, or a compound inset where an inset for a smaller sample is set into a larger inset. Gravity chucks are mechanically simpler, and are not subject to clogging or failures related to vacuum systems, which are notorious for breaking down or requiring repair. The absence of a vacuum system and rotary union allows for a more cost effective and simpler implementation.

Some more “affordable” vacuum chuck based spin coaters costing a few thousand dollars (USD) have, at least within our research institution, been unreliable and subject to repeated failure and/or down-time usually related to the vacuum chuck, vacuum system and the path between. This observation is localised and may not be a fair representation of the manufacturers of these devices.

When designing shorter term research projects such as in MSc and undergraduate studies the wait time for new equipment or for parts to repair existing equipment can elapse a significant fraction of a student’s available time. This is strongly affected by geo-bureaucratic circumstances, including the time required to organise and process funding for equipment, shipping time, customs delays, and costs. Also when working with imported commercial equipment, repair costs and logistics can result in significant down-time while waiting for a technician, shipping the instrument, or arranging finances.

While commercial builds cost a few thousand dollars, with additional sample chucks costing from $90 to a few hundred dollars (examples in SI Section 13), DIY spin coater builds can cost a few hundred dollars or even less making them a far more attractive option when cost, cost of repair and ease of repair are of primary importance.

In this paper, we present a simple, low cost 2-stage spin coater with a novel gravity chuck design. This makes use of a chuck and mask system with the full build planned around repairability, robustness and simplicity. These benefits are in part a result of bypassing the common points of failure in vacuum dependent designs.

## 2 Hardware description

**Fig. 1:**
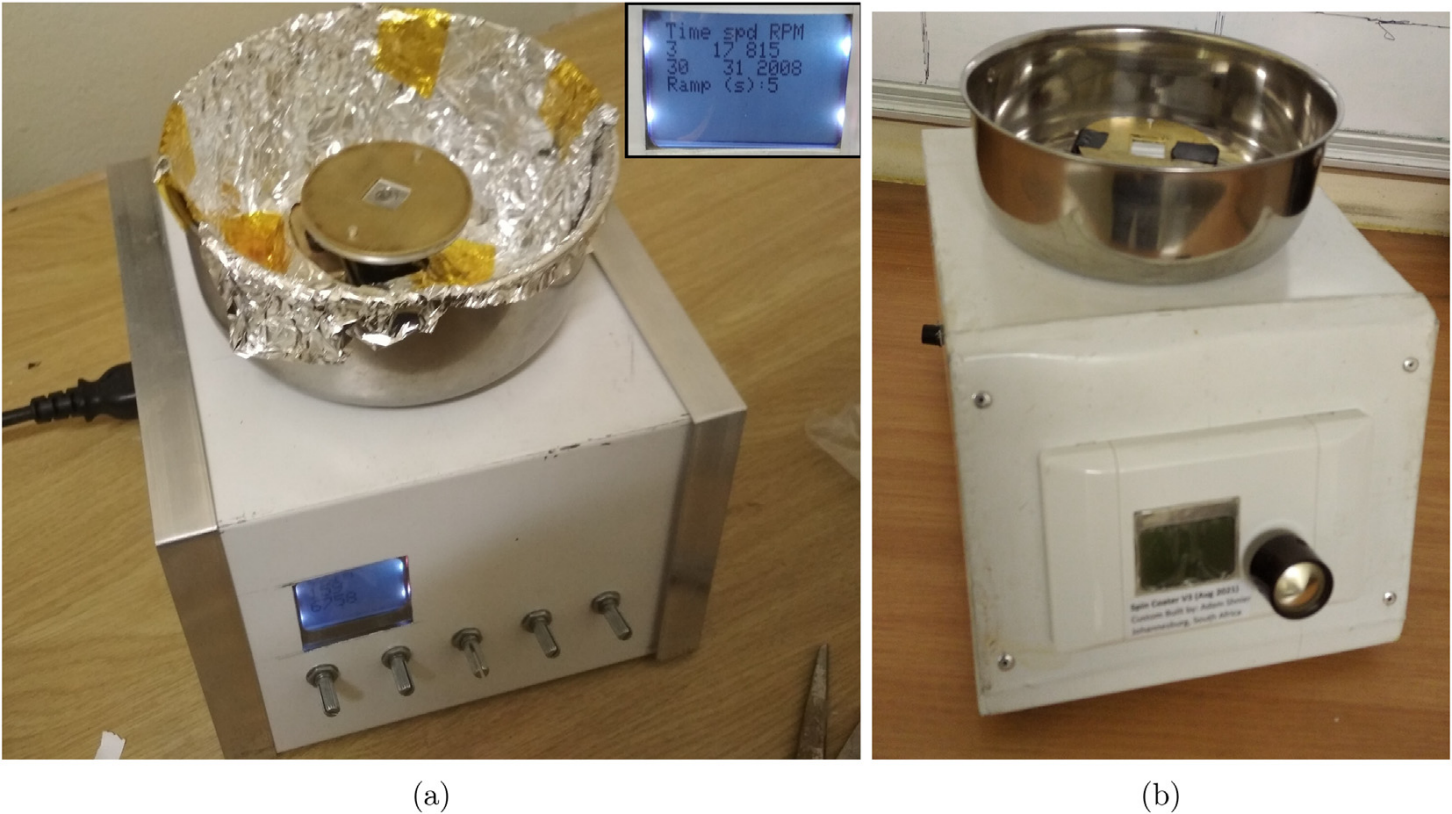
The assembled spin coaters, (a) Hardware version 2 (hV2) lined with foil and with an inset showing the pre-run display of the set run parameters, (b) Hardware version 3 (hV3) with the sample mask taped down for transport to Maseno University, Kenya.

This spin coater consists of a gravity chuck, motor, power supply, electronics, software, and the enclosure. It is a low cost device with simple circuitry built around an Arduino Nano compatible microcontroller to drive a DC motor and provide information on a Nokia 5110 display. The novelty of the chuck design lies in the plate like sample holder mask, and the round pins extending from the chuck on which the mask rests as the holding mechanism. In order to accommodate samples of different sizes, only the sample mask needs to be exchanged, in a quick tool free process. Two prototype implementations are presented in Fig. 1.

This hardware can be useful in any facility where thin films are being studied, which includes research in the following fields:

• Thin-films device fabrication, including thin film solar cells.
• The study of optical and optoelectronic properties in thin films.
• Education to show an application of centrifugal force, and the relationship between spin speed and film thickness. This spin coater can be simplified as in hardware Version 1 (hV1) (See reference [6]) for applications such as teaching motor control or optical properties of materials [7].

### 2.1 Mechanics and Hardware

*Motor*

The spin coater is driven by a permanent magnet DC motor which is mounted to the enclosure, with a rubber gasket between as a vibration dampener. In Hardware version 2 (hV2), the motor axle had an M8 thread while hV3 had a 6.35 mm smooth shaft. Some commercial spin coaters have shafts of 3 mm. Under normal operation, it is inconceivable that any issue would arise from this, yet considering the surprising feats students are capable of, the motors were chosen to have thicker shafts which cannot easily be bent. The rotation speed is motor dependent, in the case of hV2 the motor operates comfortably between 600 and 7000 rpm. At lower speeds the force exerted by the motor magnets is significant and when the PWM (pulse-width modulation) duty cycle is below 5 % (PWM 13/255, ± 500 rpm), the motor magnet and other mechanical resistive forces are very close to or exceed the force generated by the motor coils potentially stalling the motor.

*Tachometer*

**Fig. 2:**
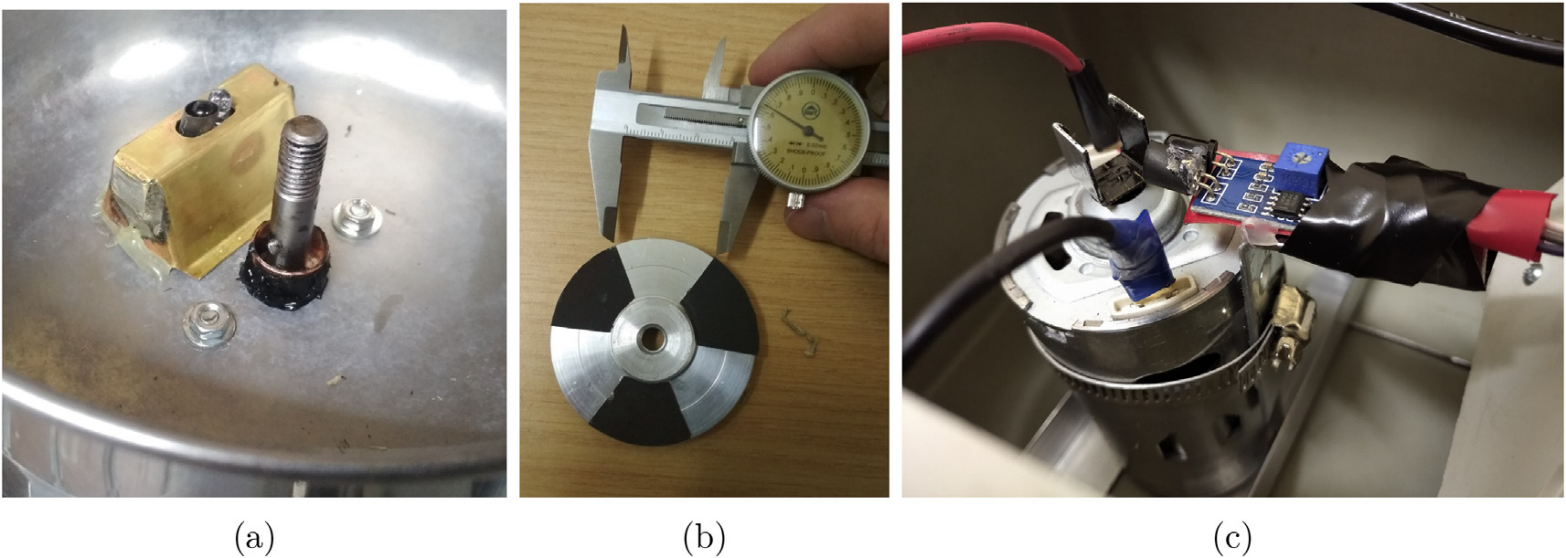
Tachometer assembly showing (a) the infra-red proximity sensor for **hV2**, (b) the reflector/absorber pattern on the underside of the chuck in **hV2**, and (c) the reflector/sensor assembly used in **hV3**.

The motor speed was read using an infra-red proximity sensor. In hV2 the underside of the sample chuck was patterned into evenly spaced alternating sections of black 1000 grit sandpaper and bare reflective aluminium, as shown in Fig. 2b. The sandpaper provides a very good light absorbing surface, with the roughness diffusing any light that could be reflected by a smooth black surface. This approach required mounting of the sensor directly under the chuck, as shown in Fig. 2a. In hV3 (Fig. 2c) this system was replaced by mounting a reflector on the small portion of shaft protruding from the bottom of the motor. This reflector was made from a brass strip bent into a “U” shape. This was painted black with the outer sides covered with a reflective aluminium tape. The infra-red sensor gain/sensitivity needs to be set such that it is not close to either providing false positive nor false negative readings. Both implementations make use of a strong reflector/absorber type contrast. This allows strong discrimination between reflective and “dark” conditions. Additionally, the sides of the infra-red receiver on the sensor was covered with black heat shrink (glued in place) to reduce the light received directly from the adjacent transmitter. This allows a higher sensitivity/gain to be set on the sensor, while preventing false positive readings from this effect. Comparing the sensor placement in hV2 and hV3, the sensor in hV2 was in the coating chamber directly under the sample chuck. This resulted in a more complicated mounting implementation requiring a protective enclosure. In hV3 the sensor mounting was entirely in the main enclosure. The tachometer assembly is included in SI documents *“Assembly of Spin-Coater hV2.pdf”* and *“Assembly of Spin-Coater hV3.pdf”*.

*Sample chuck and mask*

**Fig. 3:**
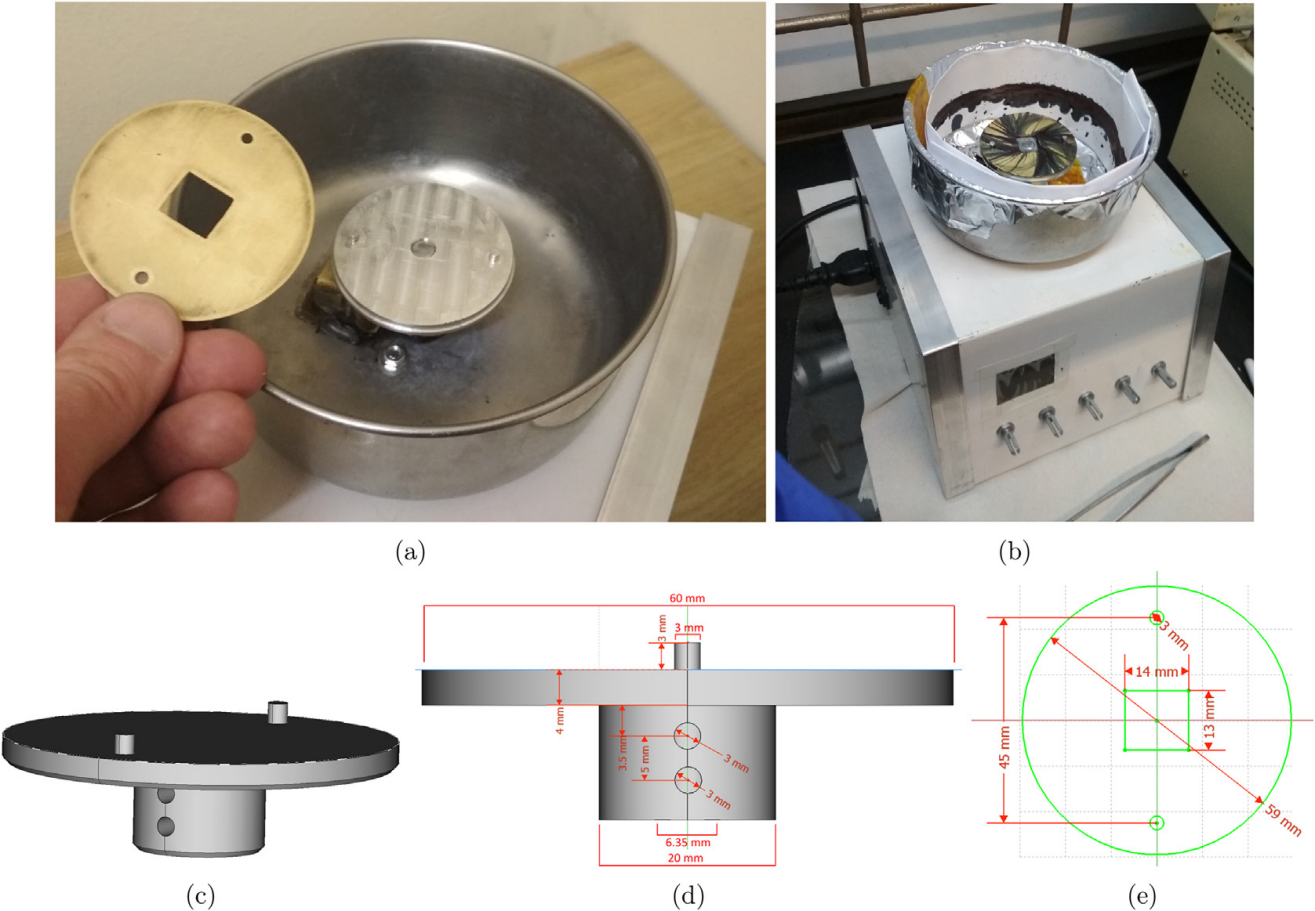
Sample chuck and mask. (a,b) hV2 chuck and sample mask, (c,d) hV3 sample chuck for mounting on the motor shaft using M3 grub screws on the side, with 3 mm pins on top. (e) Sample mask 14x13mm for hand cut “12.5x12.5 mm” samples. Design Files are included as “Chuck_smooth-shaft.FCStd” and “Masks.FCStd” drawn in FreeCAD V0.18.

The chuck design (Fig. 3) makes use of a flat top with two 3 mm pins positioned 45 mm apart. On these, an easily machined sample mask sits, allowing quick and simple interchange between sample sizes without removing the chuck, and the easy crafting of new masks for different sample sizes. The chuck was machined by a workshop technician from a cylindrical aluminium block with a CNC router to form the top with pins, and a lathe to shape the profile. hV2 makes use of a chuck with a thread tapped in the centre to fit on a motor with a threaded shaft, while hV3 has a blind hole in the chuck with 2 grub screws to fix it on a smooth motor shaft. An alternative chuck version for a motor can be made without these machining tools, as described in SI Section 3.1.

The sample mask is a 0.7 mm brass plate that has two 3 mm holes corresponding to the chuck’s 3 mm pins, along with a cut-out in the centre corresponding to the sample/substrate size used. The sample mask should be thinner than the samples to allow easy drainage of the precursor solution while coating. The 0.7 mm mask was paired with 1.1 mm thick samples, this being the thickness of the microscope slides and ITO coated glass used within our research group. The sample mask is easily exchanged, which is especially valuable in a lab where multiple users work with substrates of different sizes.

The sample chuck and mask system is ideal for substrates slightly thicker than the sample mask. Very thin samples such as a 300 μm silicon wafer risk slipping under the mask. To avoid this, they can be mounted with double sided tape on a glass slide or equivalent for spin coating. As mentioned, vacuum chuck spin coaters risk creating a curvature in ultra thin samples, and this may similarly be remedied by mounting onto a glass slide. Another good alternative for very thin samples is to use inset type chucks [8]. With these, thin samples cannot slip between the sample chuck and mask. Inset type sample holders can be made to fit the presented chuck design using holes corresponding to the pins of the hV2/hV3 chuck for easy mounting. These can be produced by either 3D printing, using a CNC router, or by gluing a sample mask to a backing plate (these have not been produced for this work).

Commercial alternatives using vacuum or gravity chucks typically require the entire chuck to be exchanged to change the sample mount [1], [9], [10], [11]. Mounting systems in open-source designs include the use of double-sided tape, vacuum chucks when using hard drive motors, gravity chucks where adjustment for sample size requires tools and measurement, and chucks using removable screws to secure samples [12], [13], [7], [2], [14]. In comparison, the chuck design presented here provides a simple, rapid, easy, and tool-free method to exchange the sample mounting configuration which takes close to 5 s to swap the sample mask.

Commercially, chucks are available at purchase prices ranging from $90 to $300. In cases where chucks can be 3D printed [8], the associated knowledge and access to a 3D printer with a fine enough print resolution is required. In the design presented, the majority of technical expertise rests in the manufacture of the chuck, while the brass masks which accommodate samples are less sensitive to machining accuracy, and are easily crafted with readily available tools and inexpensive materials (i.e. a drill, saw or tin snips, small metal files and brass sheet). This offers the advantage of lower cost, reliable access without the risk of parts being discontinued, and avoids shipping, logistics and other potential delays in favour of locally available components. [15], [16].

The need to accommodate a new shape and size of sample can occur long after the spin coater was commissioned. There is no guarantee the same individuals, technical knowledge and resources that were involved when equipment was commissioned will still be accessible. The simplicity and ease of machining a sample mask from brass plate serves as an advantage over other commercial and open source alternatives [15].

All the components are supported by, and housed in the enclosure as shown in Fig. 1. It serves to support the motor and protect the electronics in a lab environment, while the stainless steel bowl acts to contain the waste fluid from the spinning process. An enclosure can be bought or built using whatever non-flammable materials are most accessible. In our case, this was offcuts of steel barge flashing (a roofing material). Information regarding the materials for and assembly of the enclosure is provided in the SI documents *“Assembly of Spin-Coater hV2.pdf”* and *“Assembly of Spin-Coater hV3.pdf”*

### 2.2 Electronics

*Inputs*

**Fig. 4:**
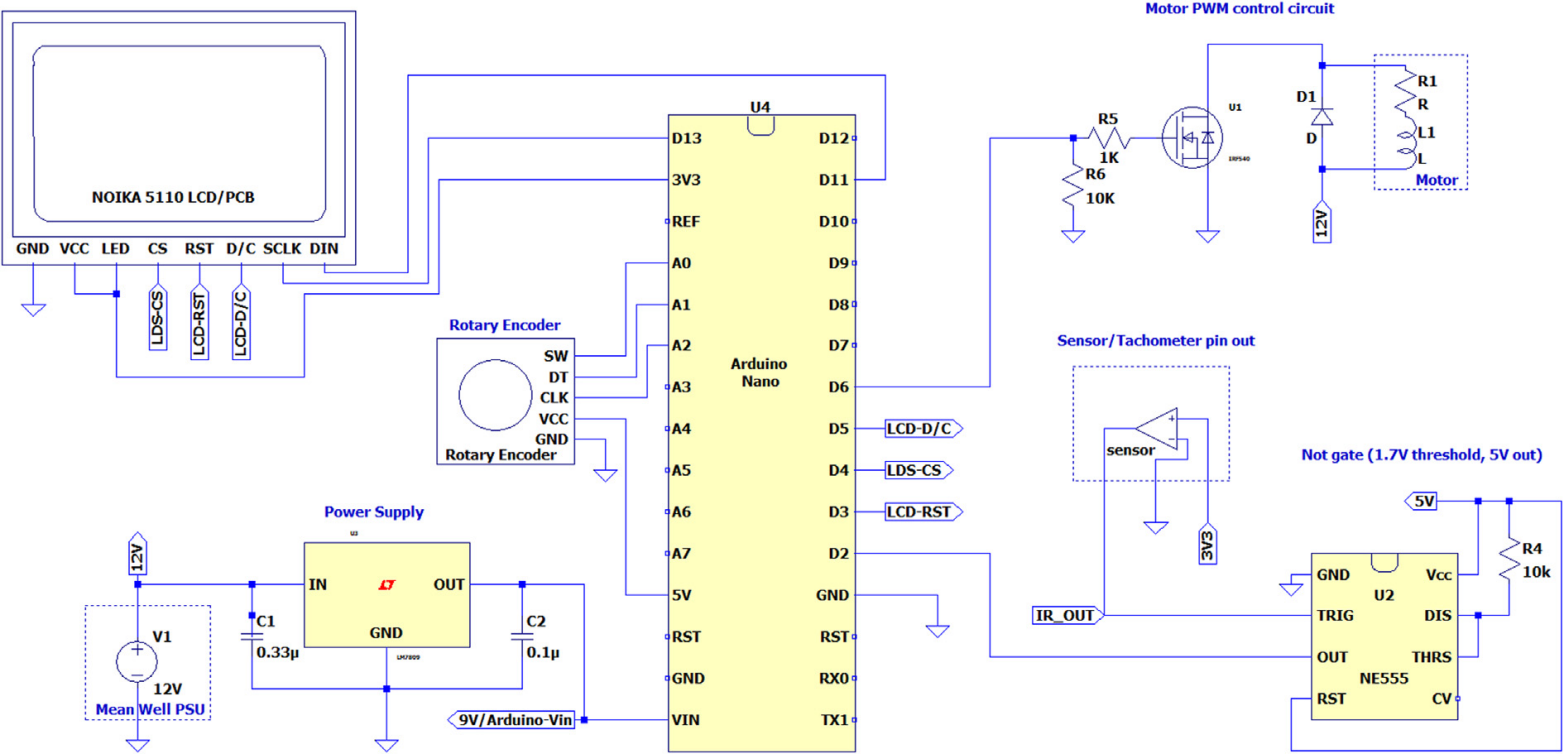
Circuit diagram for hardware version 3.

An Arduino Nano compatible microcontroller (Nano) formed the core of the electronics as seen in Fig. 4. The Nano functioned to control the DC motor with a pulse-width modulated (PWM) signal through an IRF540 n-channel MOSFET. In hardware version 2 (hV2), 50 kΩ potentiometers (pots) were used to set the spin parameters, with each sending a voltage between 0 and 5 V to the analog pins of the Nano for each parameter. Momentary contact buttons for start and reset functions were included in hV2. The pots used in hV2 provided unsTable 10-bit values (0 to 1023). The source of the instability could be related to the 5 V 3A USB power supply used for the Nano, which may additionally be affected by the power draw of components directly connected to the Arduino in our build. The instability was managed using a software based approach discussed in the software section.

**Fig. 5:**
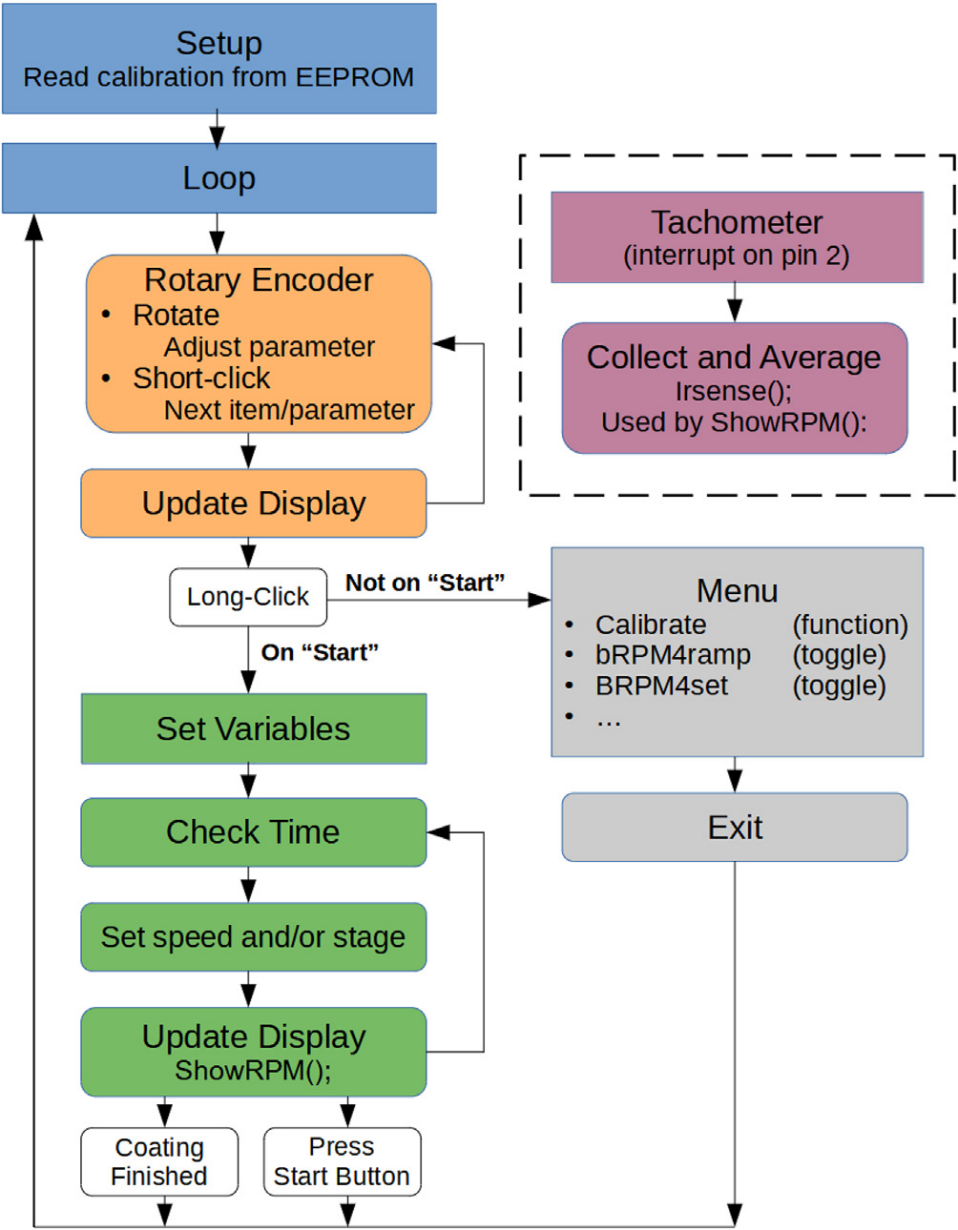
Flow diagram of software version 2.2 operation (used in hV3).

To simplify the design and side-step the instability of the values read from the pots, hardware version 3 (hV3) replaced the pots and buttons with an incremental type rotary encoder containing an inbuilt momentary contact button. This functioned using the button to cycle through parameters, while rotation of the encoder adjusted parameter values, as shown in Fig. 5. The use of a single rotary encoder replacing the 5 pots and 2 buttons simplified the enclosure assembly, and provided digital parameter control which bypasses the need to read analogue signals from pots.

*Tachometer*

The logic level voltages of an Arduino Nano are inherited from its ATMega328 microcontroller. The digital input voltages are > 3.0 V for a digital high and < 1.5 V as a low [17]. An IR-proximity sensor working on 3.3 V was connected to the Nano through a logic level converting NOT gate, with a threshold of 1.7 V and output of 5 V made using a NE555 timer. During testing of hV2, a direct connection between the sensor and Nano did not reliably trigger a digital high input. The *logic level converting NOT gate* was included to address this for the specific IR sensor board used.

*Power delivery*

The motor was powered by a 24 V PSU in hV2, and 12 V in hV3. In hV2, power was delivered to the microcontroller by a step-down converter which delivered 5.2 V to the USB port of the Nano; a small voltage drop is expected in the USB cable. In hV3, a L7809 linear regulator reduced the voltage to 9 V which was then fed into the Power in (Vin) pin of the Nano. The Vin pin on the Nano is specified to accept 7–12 V input [18] which is regulated to 5 V by a LM1117 regulator. By dropping the voltage to 9 V prior to the Vin pin, the heat which the small onboard regulator will need to dissipate is reduced, $P_{\mathrm{Heat}}=(V_{\mathrm{In}}-V_{\mathrm{Out}})\cdot I_{\mathrm{Load}}+V_{\mathrm{In}}I_{\mathrm{Ground}}$ [19]. Linear regulators reduce voltage by resistively dissipating energy as heat, while step-down/buck converters use PWM (pulse width modulation) typically followed by low pass filters to provide a reduced DC voltage at a high conversion efficiency.

Testing a 24 V PSU with the 9 V LM7809 (TO-220 package) for the hV2 configuration resulted in slow heating in a L7809 regulator, leading to thermal overload. While the heat dissipation can be accelerated with a heatsink, a step-down converter is preferable due to the higher conversion efficiency and reduced heat generation. The lower heat generation is desired as it allows the enclosure to be sealed without a need for vents or a fan. The absence of vents reduces the gradual accumulation of dust. This is of great importance in environments such as fume-hoods, where the strong air flow accelerates dust accumulation. The chemicals a researcher uses can require a spin coater to be used in a fume-hood due to OHS considerations.

### 2.3 Software and system

The software is included in SI files *“SpinCoater*_*v2.3.1(for*_*hV3).ino”* and *“SpinCoater*_*v1.4(for*_*hV2).ino”*. These operate as per the flow diagram in Fig. 5 for hV3, and Figure SI-3a for hV2.

*Setting parameters*

The spin parameters consist of the speed and time for each of the two stages (*speed 1, speed 2, time 1, time 2*) and the ramp time (*ramp*), which is the time to be allocated in total for ramping up the speed from stationary to the first speed, and between the first and second speeds. Parameters are either set using pots in hV2, or an incremental type rotary encoder in hV3. In hV2, 10-bit (0 to 1023) DAC readings are taken from 50 kΩ pots in the range 0 to 5 V. These readings were collected by averaging 5 readings, each 5 ms apart. Parameter values were updated if the new average values differed from the stored values by more than 5 units, equivalent to 24 mV. These values were converted into the spin parameters, of which the parameters with the finest increments are speed. **Speed 1** was defined by *“s1*  *=* byte(float(a[0])*242/1023)+13;”, where s1 is *speed 1*, and *a[0]* is the averaged value read from the first pot. This converts the 10-bit value averaged from the DAC reading to 8-bit (byte) value with extrema of 13 and 242, defining limits on the minimum and maximum PWM duty cycles chosen for the specific motor. This provided good parameter control with the caveat that parameter values occasionally shifted by one unit. The parameter values are fixed when a run is started, as such these values should be checked on the display prior to each operation run.

This minor instability was addressed in hV3 by utilising a rotary encoder in place of relying on DAC read signals from the pots. Each time a parameter is updated on rotation of the rotary encoder in hV3, a delay of 10 ms is observed to allow for effects such as contact bounce before reading the state of its “SW” and “DT” pins (see Fig. 4). This provided a stable, reliable means of adjusting parameters.

*Motor control*

The motor speed is controlled using a MOSFET driven by a PWM signal. This can be generated by software, switching the signal through loops, or hardware driven, using the Nano’s *analogWrite()* command. A software based PWM loop did not provide consistent speed control which was attributed to the computational processes demanded of the Nano single processing core. The most intensive being the processing and updating of the graphics display, and the hardware interrupt signals from the tachometer which trigger priority execution of the associated software function. In contrast, consistent operation of a hardware controlled PWM signal was obtained, irrespective of the other demands on the processor. The drawback of the hardware PWM is the fixed PWM frequency and discrete steps in the PWM duty cycle, limiting the accessible speeds to discrete intervals. On a Nano, hardware based PWM is limited to a byte value between 0 and 255, corresponding to a target duty cycle of 0 and 100 % respectively.

*Calibration*

Prior to the first use, the spin coater should be calibrated (speed in RPM vs duty cycle). Calibration can be accessed though the menu as per Fig. 5, which calls a function *void Calibrate()*. Once initiated, the motor speed is measured at values across the *AnalogWrite()* PWM range. The motor is allowed time to get to speed, followed by sampling the tachometer reading and averaging 5 values (hV2), followed by time idle between each speed. The result for each PWM duty cycle is written to the Nano’s EEPROM non-volatile memory.

*Bypassing RPM calibration*

Operation can rely either entirely on the PWM duty cycle, or on RPM values as determined by the menu parameter *bRPM4ramp*. When *bRPM4ramp* is set to *True*, the speed will ramp linearly with time using the RPM calibration values. When set to *False*, the duty cycle will ramp linearly with time. In hV2, “bRPM4set” toggles whether the speeds are set using RPM or PWM values, while hV3 allows the speed to be set by either at the pre-run display, where parameter are displayed and set.

*Tachometer*

During operation the tachometer code relies on system interrupts to measure the time between successive transitions of the infra-red sensor’s signal from low to high. The motor used in hV2 will not exceed 8000 rpm (7.5 mS per revolution), with 3 interrupts per revolution this leaves 2.5 mS between transitions. The interval between interrupts is stored when successive interrupts occur slower than 2 mS apart. This minimum interval prevents multiple readings for a single transition. When the display refreshes, it calls the function *showRPM()* which averages the last 18 intervals to provide the rotational speed.

*Boost function*

The DC motors used require more torque to overcome the resistance to rotation when the motor is stationary compared to when it has some inertia. To allow the motor to run at lower duty cycles, a “boost function” was implemented in hV3. The “boost function” increases the PWM duty cycle for 50 ms to generate additional torque in the motor. The activation criteria for the boost function requires that the tachometer reads 0, and that it is between 50 and 500 ms from the start of the spin program.

Should the tachometer ever fail and be unable to provide the microcontroller with readings, the spin coater can continue to operate provided the boost function is toggled off. This serves to reduce the risk of a user facing unusable equipment while plans are made for hardware inspection and repair.

*Modularity*

The circuity, motor and tachometer are not specifically paired, allowing easy substitution of these components for repairs and modification. A replacement motor can be easily set-up by running the calibration function accessible in the menu.

There are both DIY and commercial spin coaters available offering more advanced software. Some of these include features such as active speed correction using PID control. Many available spin coaters operate with 2 stages, while some commercial and DIY devices allow for a larger number stages in the coating process. The code written for this project provides 2 stages and an easy-to-use interface.

***Design files***

## 3 Design files summary

**Table:** 

**Design filename**	**File type**	**Open source license**	**Location of the file**	
Shnier2022-SpinCoater-SI.pdf	PDF	SHL-2.1	Available with the article	
SpinCoater_parts-list.xlsx	Excel	SHL-2.1	http://doi.org/10.17632/y84tbnfsj4	
Chuck_smooth-shaft.FCStd	FreeCAD	SHL-2.1	http://doi.org/10.17632/y84tbnfsj4	
Chuck_threaded-shaft.FCStd	FreeCAD	SHL-2.1	http://doi.org/10.17632/y84tbnfsj4	
Electronics Spin hV2 20211129.zip	LTSpice	SHL-2.1	http://doi.org/10.17632/y84tbnfsj4	
Electronics Spin hV3 20220916.zip	LTSpice	SHL-2.1	http://doi.org/10.17632/y84tbnfsj4	
Video of operation (hV2)	Video	MIT	http://doi.org/10.17632/y84tbnfsj4	
Assembly of Spin-Coater hV2.pdf	PDF	SHL-2.1	http://doi.org/10.17632/y84tbnfsj4	
Assembly of Spin-Coater hV3.pdf	PDF	SHL-2.1	http://doi.org/10.17632/y84tbnfsj4	
SpinCoater_v2.3.1(for_hV3).ino	Arduino Sketch	MIT	http://doi.org/10.17632/y84tbnfsj4	
SpinCoater_v1.4(for_hV2).ino	Arduino Sketch	MIT	http://doi.org/10.17632/y84tbnfsj4	
SpinCoater_not-gate.zip	LTSpice/ various	SHL-2.1	http://doi.org/10.17632/y84tbnfsj4	

*Design files descriptions*

• *Shnier2022-SpinCoater-SI 20220914.pdf* provides additional aspects of and concepts around the spin coater. This includes a list of commercial and DIY or open-source spin coaters.
• *SpinCoater*_*parts-list.xlsx* is an Excel spreadsheet containing a more detailed list of parts used for spin coaters hV2 and hV3 with the enclosure parts included.
• *Chuck*_*smooth-shaft.FCStd* and *Chuck*_*threaded-shaft.FCStd* contain the sample chuck dimensions the 6.35 mm shaft variants of hV3 and the M8 threaded shaft of hV2 respectively. The threads for the shaft is represented with an 8 mm hole in hV2 and the M3 tapped thread for grub screws of hV3 are represented by two 3 mm holes in the CAD drawings.
• *Electronics Spin hV2*_*20211129.zip* and *Electronics Spin hV3*_*20210916.zip* are LT spice schematics showing the connectivity of the electronics including the motor, IR sensor, LCD display and rotary encoder.
• *Video of operation (hV2) Shows a typical use case for spin coater hV2.*
• *Assembly of Spin-Coater hV2.pdf* and *Assembly of Spin-Coater hV2.pdf* detail the build processes and techniques for the corresponding spin coaters.
• *SpinCoater*_*v2.3.1(for*_*hV3).ino* and *SpinCoater*_*v1.4(for*_*hV3).ino* containing the Arduino code designed for the corresponding spin coaters.
• *SpinCoater*_*not-gate.zip* contains the LTSpice schematic for the NOT gate along with images of the simulations run on the circuit.

***Bill of materials***

## 4 Bill of materials summary

**Table:** 

**Designator**	**Component**	**Qty**	**Unit USD**	**Total USD**	**Source of materials**	**Material type**
	**Common Electronics - Total**			$20.79		
COM	Nokia 5110 LCD Module (15M8247)	1	$10.99	$10.99	mantech.co.za	semiconductor
COM	Infrared Obstacle Avoidance Sensor	1	$0.84	$0.84	pishop.co.za	semiconductor
COM	NE555 timer	1	$0.23	$0.23	mantech.co.za	semiconductor
COM	Resistor 10 k Ohm	3	$0.04	$0.12	mantech.co.za	other
COM	Resistor 100 Ohm	1	$0.04	$0.04	mantech.co.za	other
COM	Resistor 1 k Ohm	1	$0.04	$0.04	mantech.co.za	other
Motor control	MOSFET IRF540	1	$3.00	$3.00	mantech.co.za	semiconductor
Motor control	Rectifier diode DO41 1A 400 V	1	$0.01	$0.01	mantech.co.za	semiconductor
COM	PROTOTYPING PCB 2.54 PITCH (14M1306)	1	$1.54	$1.54	mantech.co.za	composite
COM	Female headers (14M6379)	3	$1.02	$3.06	mantech.co.za	composite
COM	JUMPER LEAD PLUG-SOCKET 200 mm	1	$1.68	$1.68	mantech.co.za	composite
COM	WIRE SOLID 0.34 1.6MM 250 mm (red/black/yellow)	3	$0.14	$0.42	mantech.co.za	composite
COM	Hex Brass M3x10mm Stand off	4	$0.12	$0.47	mantech.co.za	metal
COM	M3 nut	4	$0.02	$0.07	mantech.co.za	metal
	**hV2 Specific Electronics - Total**			$74.66		
PWR-hV2	Dual USB Output 6–24 V To 5.2 V 3A Step Down Converter	1	$2.68	$2.68	pishop.co.za	semiconductor/other
PWR-hV2	Mean Well Power supply 24 V $100\text{W}{\;}^{*1}$	1	$26.18	$26.18	mantech.co.za	other
hV2	DC motor 24 V 6000 rpm XD-3420	1	$35.73	$35.73	amazon.com	other
UI-hV2	Buttons (momentary contact) 72M4324/72M2826	2	$1.67	$3.33	mantech.co.za	other
UI-hV2	Potentiometer Linear 17MM 50 Kω PCB	6	$1.12	$6.74	mantech.co.za	other
	**hV3 Specific Electronics - Total**			$54.68		
hV3	Motor JAK Motor 6-18VDC 79 W RS-997SH-7013F	1	$16.64	$16.64	mantech.co.za	other
PWR-hV3	Mean Well Power supply 15 V $100{\text{W}}^{*2}$	1	$33.06	$33.06	mantech.co.za	other
PWR-hV3	REG FIX POS TO220 9 V 1A - L7809CV	1	$0.26	$0.26	mantech.co.za	semiconductor
PWR-hV3	Capacitor Ceramic Disc 330nF	1	$0.26	$0.26	mantech.co.za	other
PWR-hV3	Capacitor Ceramic Disc 100nF	1	$0.24	$0.24	mantech.co.za	other
UI-hV3	Rotary Encoder Keyes - KE0053	1	$3.22	$3.22	mantech.co.za	other
-hv3	Brass sheet 0.9 mm, 40x10mm	1	<$1	<$1	metalcentre.co.za	metal
	**Chuck and Mask - Total**			$5.12		
C&M	Aluminium rod, ϕ = 65 mm, L = 55 mm	0.5	$4.07	$2.04	metalcentre.co.za	metal
C&M	Brass plate 65x65x0.7 mm	3	$1.01	$3.03	metalcentre.co.za	metal
C&M-hV3	Grub screw M3	2	$0.03	$0.05	mantech.co.za	metal

* The enclosure components are detailed in the SI document *SpinCoater*_*parts-list.xlsx*.

*1 The motor has a rated locked-rotor current of 3.37 A at 24 V (81 W).

*2 This motor is currently running off a 12 V 2A PSU and comfortably reaches 5200 rpm.

**COM:** Electronic components common to hV2 and hV3.

**UI:** User interface.

**PWR:** hV2 and hV3 specific power delivery related components.

**C**&**M:** Chuck and mask related materials.

**-hV2, -hV3:** Components specific to hV2 or hV3, mostly used in combination with other categories.

## 5 Build instructions

The spin coater build consists of an enclosure, motor, and the electronics which include the power supply. Safety should be the first priority when building and operating equipment. A selection of considerations are listed below, with some additional precautions and best practices in the SI Section 10. This is intended as informative and is not comprehensive:

• When working with electricity all connections and wiring should be done while the power is off and the device is disconnected from mains.
• Capacitors, such as those present in power supplies (PSUs) store charge even after the PSU is disconnected from mains power. These can discharge explosively if shorted.
• Soldering involves high heat, care should be take to avoid burns while good ventilation is required for the fumes produced from solder, fluxes, and potentially but hopefully not, your components.
• When working with a motor keep the area clear of anything that could get caught by the motor such as loose clothing, jewellery, or hair.
• The spin coater’s chuck and sample mask should not be touched when rotating. At speed the sample mask could potentially mimic a rotary cutting tool.

The components needed for the power supply depend on the chosen motor.

• A 12 V motor should be paired with a 12 V PSU with a sufficient power rating to supply the motor. The voltage of the PSU also affects how power can be supplied for the microcontroller. The microcontroller steps down the input power from the Vin pin using a linear voltage regulator which dissipates heat proportional to the voltage difference between the input and output voltages. To reduce the heat dissipation on the board itself the 12 V was lowered to 9 V using a LM7809 linear voltage regulator to supply the microcontroller’s Vin pin.
• For the 24 V motor and PSU in hV2 there is a much larger voltage difference making a linear voltage regulator less suitable due to heating concerns. For this a buck converter with a 5.2 V USB output was used to power the Nano through its USB port.

The connections for the electronics are as per the circuit diagram in Fig. 4. The following considerations were made in assembly:

• Use of female headers on the PCB to allow detachable connections for the *Arduino Nano compatible microcontroller*, *rotary encoder*, *display* and *tachometer sensor*. These connections were made with ribbons of jumper leads.
• For the *power in* and *motor power* connections PCB mounted screw terminals were soldered to the PCB.
• A DIP8 socket was soldered to the PCB to house the NE555 timer chip.
• The remaining connections were soldered in place.
• The use of removable components allows for easier trouble-shooting as components can be disconnected or exchanged for testing.
• The choice of infra-red proximity sensor will affect whether the logic converting circuit is needed for signals to be reliably read by the Nano. For proximity sensors which provide 5 V logic signals the *NOT gate* sub-circuit will not be needed. Ideally 3.3 V logic signals should work reliably directly connected to the Nano, yet with the sensor tested in our application this was not the case. It is suggested that 3.3 V proximity sensors should be tested in a bread board to assess the need for logic level converting circuit, such as the *NOT gate* sub-circuit.

The enclosure can be built in innumerable ways such that the motor is supported, the electronics are protected, and a chamber is present for collecting the waste liquid thrown off the sample during coating. Enclosure build instructions are available as follows:

• See the SI document, *“Assembly of Spin-Coater hV2.pdf”*
• See the SI document, *“Assembly of Spin-Coater hV3.pdf”*
• Instructions for hV1, which makes use of a simpler design is available at [6]

## 6 Operation instructions

The spin coater operation consists of loading a sample, setting parameters, and initiating the run. Appropriate safety precautions should be observed when working with spin coaters. A few prominent safety concerns are mentioned here, with the caveat that the list is by no means exhaustive. Precursor solutions used in spin coating are expected to be aerosolised and/or evaporated to some extent. It is necessary to take appropriate precautions such as working in a well ventilated area, in a fume-hood, or in a glovebox as determined by the chemicals in use. The spin coater includes a rotating motor, spinning chuck, and sample, thus appropriate precautions include not wearing loose clothing, and keeping anything that could potentially get caught by the moving parts clear of the space. Protective eyewear is essential, as the device does not include a cover for the enclosure-bowl. The spin coater chuck and mask should not be touched while in motion. Some additional information on best practices and safety precautions are offered in the SI Sections 10 and 11.

1. Ensure that the inner part of the enclosure-bowl is lined with aluminium foil
2. Make a protective paper ring from strips of A4 paper cut lengthways by joining the paper with folds, masking tape or staples. Place the paper ring inside the enclosure-bowl along the outer edge (see Fig. 3b). This collects most of the excess precursor from the spin coating process. This can then be changed after each set of samples to reduce the risk of sample cross-contamination and prevent gunk build-up in the coater.
3. Ensure the desired sample mask is in place, or change the mask for the substrate being coated.
4. Turn on the spin coater at the power switch.
5. (Calibration, first use) If the spin coater has not been previously calibrated, load a test or old substrate/sample and run the built in calibration program. Calibration is best run with a sample and sample mask in place to provide conditions similar to standard operation.
  Access the menu (described below) and cycle through the options as explained on the screen. Select calibrate and allow the calibration to run. It will cycle through the PWM parameter values up to 255, saving an average RPM for each value as it progresses (this takes about 2 h on hV3).
  *hV2.* To access the menu on hV2, long press the red start button.
  *hV3.* To access the menu on hV3, long press the rotary encoder with any item other than “Start” selected.
6. Adjust the spin coating parameters as appropriate for the hardware variant:
  *hV2.* Adjust the 5 pots, each of which correspond to a single parameter. There will be a “>” in front of the parameter while it is being adjusted.
  *hV3.* Use short presses on the rotary encoder to scroll through parameters, the selected item is indicated with a “>” on the LCD display. Rotate the rotary encoder to adjust the parameters.
7. (Optional) Warm up the motor by running the spin coater for 2 min. This affects the motor grease viscosity and coil windings resistance which can affect motor speed for a specific PWM duty cycle. A comparison of the tachometer reading and spin parameters is valuable to assess whether the motor needs to be warmed up, and as an assessment of the calibration values.
  *hV2.* Press the red button to start a run.
  *hV3.* Long press on the rotary encoder with “Start” selected to start a run.
8. Place a substrate in the cut-out of the sample mask.
9. Place precursor solution on the substrate using a dropper or micro-pipette.
10. Start the program as described in Step 7
11. (Optional) To stop the spin coater early press the red button (hV2), rotary encoder (hV3).
12. Remove the coated sample.
13. The spin coater returns to the parameter editing mode after a run and can be started again as done in Step 7, using the red button.
14. Remove and dispose of the contaminated paper ring from Step 2 once finished.
15. Clean and store.

## 7 Validation and characterization

Direct and indirect indications of spin coater performance were assessed. This includes the stability of the calibration speed vs duty cycle calibration data, the effect of repeated operation, and the analysis of thin films fabricated with hV2 by means of highly sensitive measurements.

**Fig. 6:**
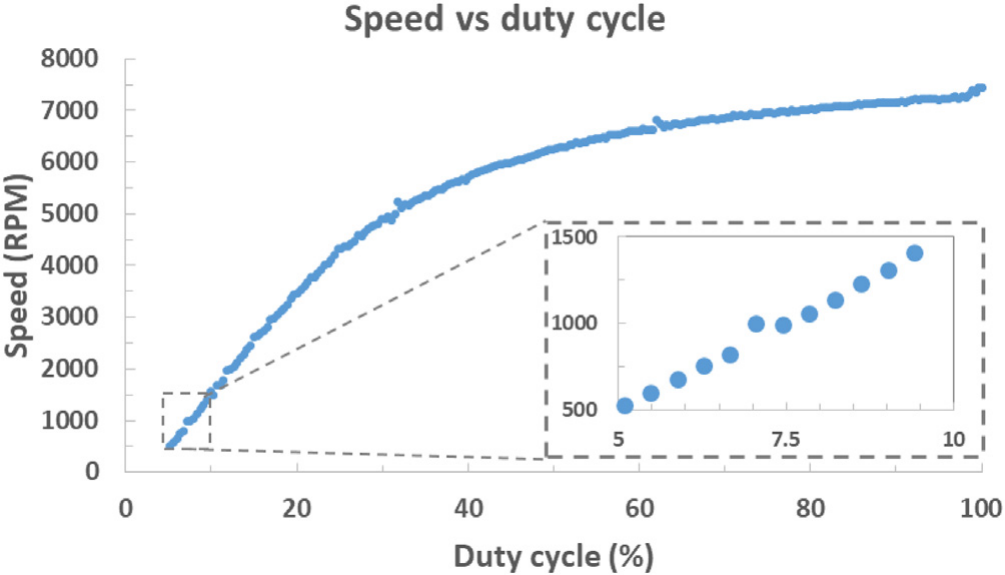
The relationship between motor speed and duty cycle for the 24 V DC motor controlled through a n-channel MOSFET with an Arduino Nano equivalent microcontroller as used in hV2.

The calibration function was run on the spin coater. Once completed, the speed vs. PWM parameter data was read from the spin coater’s EEPROM, then converted into speed vs duty cycle for Fig. 6. This was fitted with a 6th order polynomial, $R^{2}=0.9997$, Residual std. error =31.3, Figure SI-14. The magnitude residual std. error is dependent on the choice of motor and power supply stability. At higher speeds the PWM control allows finer increments as the calibration curve’s gradient decreases. This is represented by the mean step in the RPM values of 81 below 3000 RPM, and 37 between 4000 and 6000 RPM. This imposes a granular selection of spin speed and informs on the step size limitations of the Nano’s hardware based PWM control.

**Fig. 7:**
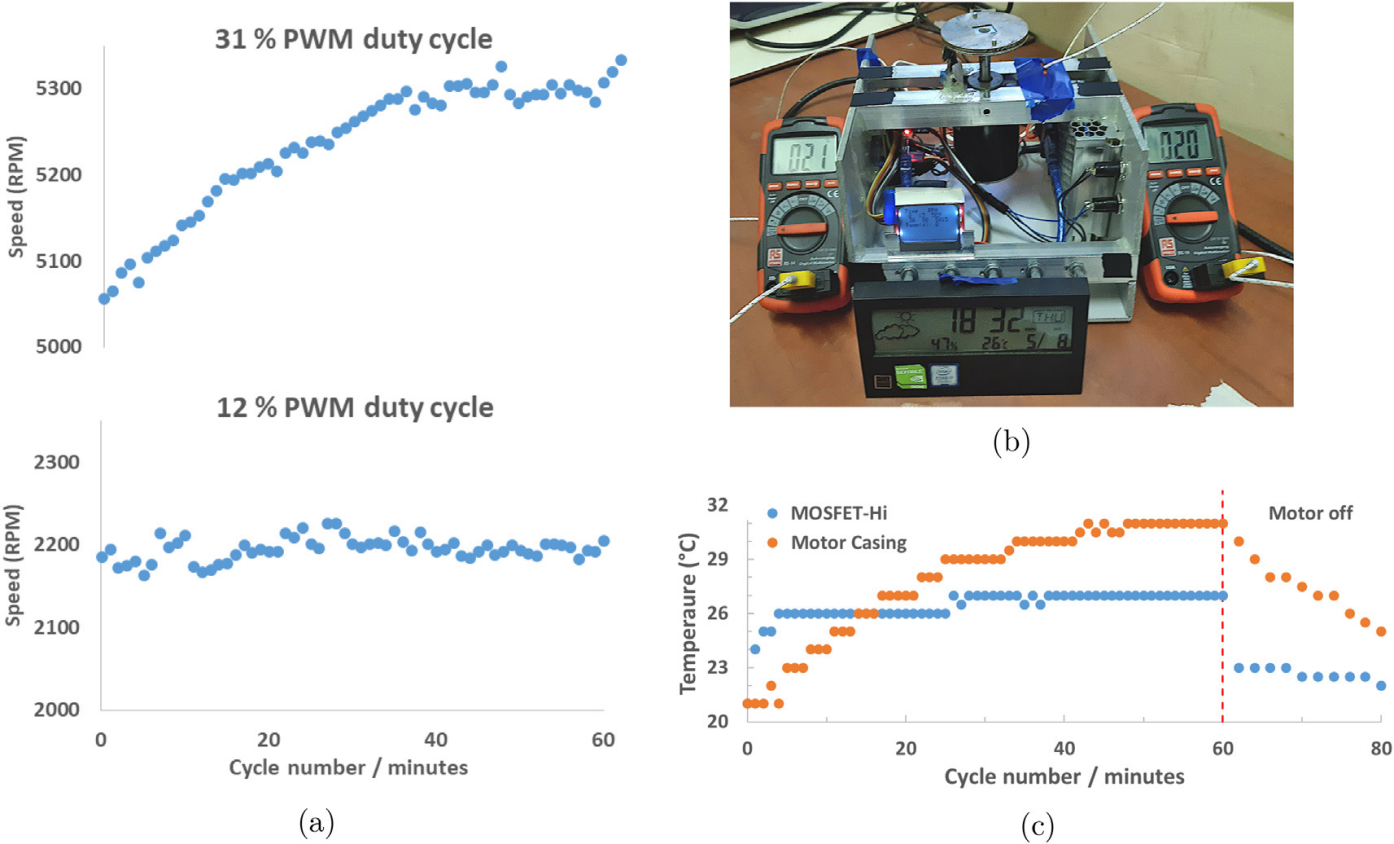
The effect of extended operation observed through cycling of 30 s on, 30 s off for hV2. (a) Motor speed as a function of cycle number with the at PWM duty cycles of 12 and 31 % (nominally 2200 and 5000 rpm respectively). (b) The hV2 spin coater with thermocouples attached for collection of temperature data. (c) Motor housing and MOSFET temperature as a function of cycle number at a 31 % duty cycle.

In testing the effect of extended operation of the spin coater, a cycling experiment was conducted. For each cycle, the spin coater was operated at a set duty cycle for 30 s followed by 30 s idle. The spin coater was started on the beginning of each minute over an hour. Multiple readings of the motor speed (> 5) were taken and averaged for each data point during the 30 s active period (Fig. 7a). This was carried out with the enclosure fully assembled, as per standard operation.

In a separate instance, K-type thermocouples were placed on the back of the MOSFET used for PWM control, and on the top of the motor housing. The thermocouples were monitored using digital multimeters with a resolution of 1 °C, as shown in Fig. 7b. In cases where the multimeter reading was unstable between 2 consecutive integers, the average was taken leading to some values with a half. The cycle number was denoted by *n*; From time ((n-1)·60 s) to (((n-1)·60 s)+30 s) the motor is running, after which the motor is idle until the $n^{\mathrm{th}}$ minute. The MOSFET-Hi temperatures were taken at ((n-1)·60 s) + 30 s while the MOSFET-low temperatures (Figure SI-15) were taken at the end of the cycle on the minute mark (n·60 s). The motor casing/housing temperature was recorded as the maximum stable value over the 1 min period of the cycle, Fig. 7c.

In Fig. 7a, the spin coater (hV2) motor speed remains reasonably steady with cycling at around 2200 rpm while operating at a duty cycle of 12%. When this is increased to a duty cycle of 31%, the motor speed gradually increases from 5050 to 5300 rpm, this constitutes a 5% increase in speed, after an hour of cycling. This was suspected as an effect of heating in either the motor or the MOSFET controlling the motor. The temperatures of both components were tracked using the same 30 s on 30 s off cycling approach (Fig. 7c). The MOSFET was quick to stabilise between 26–27 °C during cycling at a 31% duty cycle, while the temperature of the motor housing rose in a similar trend to the speed in Fig. 7a (see Fig. 7c).

To support assignment of motor temperature as the cause of the speed increase, we consider the motors no-load speed $n_{0}$ as a suitable indicator for the trend in operational speed. This neglects the effect of the weight of the sample chuck which is minimal. The relation is given in Eq. 1 where $K_{E}$ is the voltage constant of the motors permanent magnets, and $\alpha_{m}$ is the permanent magnets temperature coefficient [20], [21]. For small changes in $\alpha_{m}\Delta T$ $(\alpha_{m}\approx -0.002^{\circ }C^{-1},<40^{\circ }C),n_{0}$ can be approximated to increase linearly with increasing ΔT.

(1) $$n_{0}\propto \frac{V_{T}}{K_{E}}\mathrm{where}K_{E}=K_{E}^{0}[1+\alpha_{m}\Delta T]\Rightarrow n_{0}\propto 1/(1+\alpha_{m}\Delta T),\alpha_{m}<0$$

With Eq. 1 in consideration, the increase in motor speed was attributed to an increase in motor temperature during cycling at a the higher PWM duty cycle of 31 %. The motor used in hV2 had a sealed housing which does not allow air to pass through for cooling of the motor. It is recommended that either a motor be used that generates less heat or one which has a better heat dissipation. To accommodate this dynamic behaviour in hV2, the PWM duty cycle set point can be monitored and modified in cases of extended operation to minimise the error in spin speeds. Alternatively a software based correction can be implemented.

### 7.1 Application example

With the purpose of a spin coater being the fabrication of thin films, such films were fabricated on hV2. These were then assessed using characterisation techniques sensitive to film quality.

Polymer blend thin films were prepared using the hV2 spin coater, followed by surface measurements using atomic force microscopy (AFM). A precursor solution was used containing 20 mg/ml of PBDB-T:PC71BM (1:1 ratio) in chlorobenzene. The solution was stirred overnight at 40 °C. A dropper was used to cover a clean glass substrate with the solution followed by spin coating at between 1000 and 5000 rpm for 60 s.

**Fig. 8:**
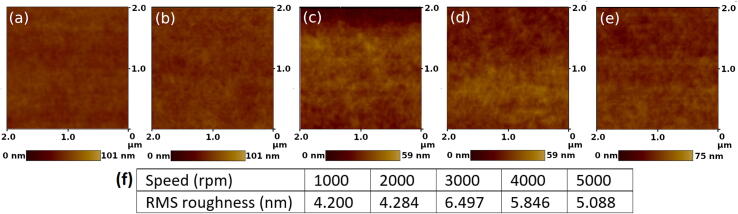
Tapping mode AFM of PBDB-T:PC71BM (1:1) thin films, spin coated for 60 s at various speed. (a) 1000 rpm, (b) 2000 rpm, (c) 3000 rpm, (d) 4000 rpm, (e) 5000 rpm (f) Root-mean-square (RMS) roughness for the various spin speeds determined across lengths scales between 2 to 5 μm.

The AFM measurements and roughness of the thin films are presented for various spin speeds in Fig. 8. The roughness appeared quite modest and consistent across the measurements. The roughness was lower at 1000 and 2000 rpm, highest at 3000 rpm and decreased thereafter. In a line-trace of the 3000 rpm sample (Figure SI-16) a maximum peak to valley height of 11 nm was observed which is comparable to the 8 nm over a 5 μm length for PCBM in literature [3]. Differences in the viscosity and concentration of the polymer blend solution, and the boiling point of the solvent used will have a strong influence on the film topography and roughness, yet these measured values are sufficient as a positive reflection on the hV2 spin coater.

X-ray reflectometry (XRR) measurements provide an indication of film quality. XRR demands high quality films with a roughness of no more than a few nanometres for the observation of Kiessig fringes. With this in mind PMMA thin films were coated on a glass substrate at 4000 rpm using hV2 with a 0.5 or 1 wt% solution of PMMA (PMMA Sigma–Aldrich 445746). The samples were dried for 20 min at 60 °C then annealed for 60 min at 95 °C in an Ar filled annealing chamber. XRR measurements were then taken on a Bruker D8 Discover with Goebel mirror and 2 bounce Ge (022) monochromator. The data was fitted using LEPTOS (Bruker AXS, V7).

**Fig. 9:**
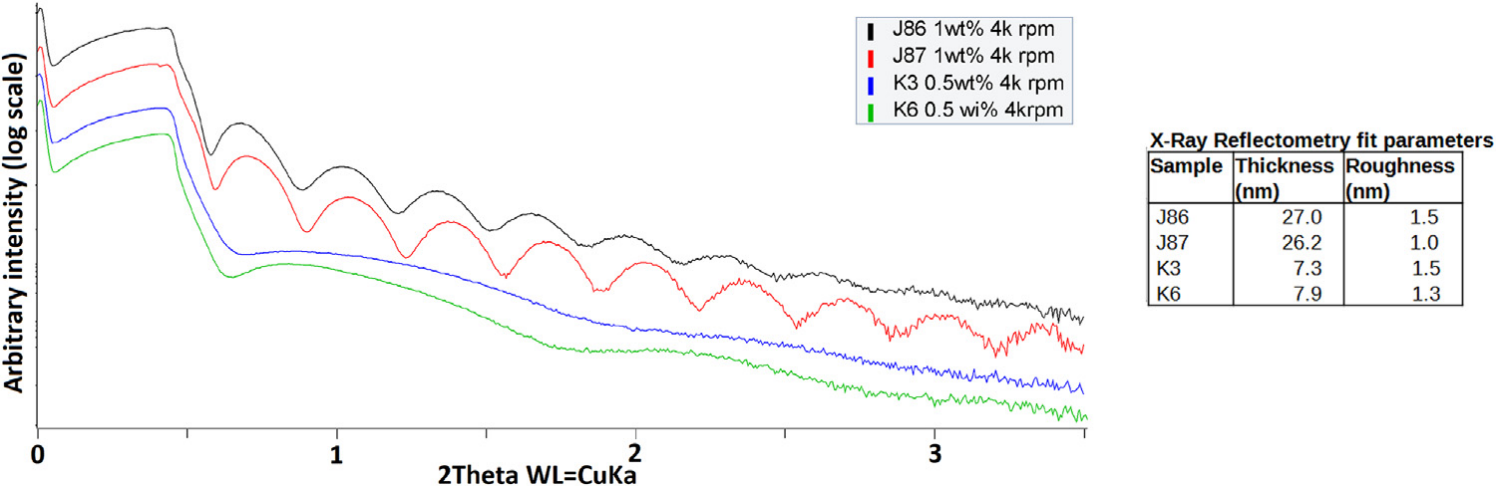
X-ray reflectometry measurements on PMMA spin coated films produced using hV2 and collected on a Bruker D8 Discover.

The measurements provided exceptionally good XRR patterns for samples prepared using spin coating, as seen in Fig. 9. Due to the low electron density and small volume of the PMMA films its density information was obscured by that of the substrate. The density of the PMMA films was thus fixed to a literature based value of 1.18 g/cm^3^ [4], [22]. While this assumption reduces the absolute accuracy of the modelled thicknesses, it increases the stability of the determined thicknesses, thus improving the relative accuracy between measurements, which is valuable for the following comparison. The thicknesses for the samples made from the 1 wt% precursor were 27.0 ± 0.3 and 26.2 ± 0.3. For the 0.5 wt% precursor thickness were modelled to 7.3 ± 0.2 and 7.9 ± 0.2. Comparable values were observed for the resulting films within each precursor weight percent solution, the slight variations observed may have contributions from minor variance in the application of the precursor to the substrate, and the wait time between this application and the start of the spin coating program.

### 7.2 Capabilities and limitations:

• Quick to change samples and sample masks
• 600–7000 rpm
• 2-stage spin program
• Max ϕ 45 mm sample size limited by the distance between pins on the chuck
• Sample masks unsuitable for ultra-thin samples
• In-built speed calibration program
• Absence of a speed correction algorithm
• Intuitive controls and display

### 7.3 Discussion and future work

The spin coater design presented provides good functionally and ease of use. hV2 as presented has survived use in a multi-user research lab in South Africa since 2019, while hV3 has been in a Kenyan research lab since 2021, without a need for repair or maintenance, exceeding the performance of at least some more costly commercial alternatives. Accommodating additional sample sizes by cutting a new mask from brass sheet is manageable by any individual competent with hand tools. This spin coater is cost effective, and allows the fabrication of high quality thin films. This makes it ideal for research groups where a reliable, repairable product is needed on a minimal budget. The cost and maintenance aspects of open-source hardware can offer utility to low budget institutes and groups where the commercial equivalent may be prohibitively expensive or otherwise inaccessible. Commercial alternatives using motors with less internal resistance provide smoother operation at low speeds compared to the motors used in hV2 and hV3. Since this is entirely dependent on the motor, this can be upgraded in future versions to close this gap.

Many of the design considerations implemented or features avoided were inspired by the authors experience (direct and indirect) with failure points and repairs of commercial spin coaters and their related vacuum systems. This includes precursor solution clogging vacuum chucks, various problems with vacuum pumps for which vacuum pumps are notorious [1], motor problems, tachometer issues causing glitches in speed regulation, and precession due to a slightly bent motor shaft among other issues.

The motor in hV2 showed gradual heating at higher PWM duty cycles. This is attributed to a closed motor housing design which leads to heat retention. In response to this, hV3 instead uses a motor with an air cooled housing.

Speed correction could be implemented to address the effects of changes in conditions, such as motor temperature on the spin speed, a simple means of speed correction would be to adjust the motor speed towards a target based on the tachometer reading. With the PWM duty cycle set by a byte value, a threshold is required to avoid constantly cycling between two adjacent speeds. The threshold can be defined relative to the step size between consecutive speed calibration values closest to the target speed, this accommodates larger steps at lower speeds and smaller steps at higher speeds consistent with Fig. 6. For more advanced speed correction Arduino libraries are available for implementation methods such as PID control [23].

To reduce the size increments of accessible speeds at lower duty cycles, effectively continuous PWM steps can be implemented. This can be achieved by using software controlled PWM. Initial tinkering showed inconsistent speed control when using software based PWM where the same microcontroller was also handling the display and tachometer. This could be easily remedied with a dedicated microcontroller to drive the software based PWM at the expense of additional cost and complexity. The finer speed increments could also benefit any speed correction implementations.

## CRediT authorship contribution statement

**Adam Shnier:** Conceptualization, Methodology, Software, Writing – original draft, Investigation. **Francis Otieno:** Investigation. **Caren Billing:** Supervision, Writing – review & editing. **Daniel Wamwangi:** Supervision, Resources, Writing – review & editing. **David G. Billing:** Supervision, Resources, Writing – review & editing.

## Declaration of Competing Interest

The authors declare that they have no known competing financial interests or personal relationships that could have appeared to influence the work reported in this paper.
