# Supplementary material for: Robust Arduino controlled spin coater using a novel and simple gravity chuck design
Source: HardwareX. 2023 Apr 17;14:e00422. doi: 10.1016/j.ohx.2023.e00422 (PMC10172839; doi:10.1016/j.ohx.2023.e00422)
Supplement: Supplementary file 1 [file mmc1.pdf]

Supplementary information for: Robust Arduino controlled spin coater using a novel and simple gravity chuck design
(Version 13 Sept 2022)

Contents

1 Electronics for hV2 **2**
1.1 Assembled circuit 3

2 Software flow diagram for hardware versions 2 and 3 (software versions 1.4 and 2.3) **4**

3 Variations on sample chuck design **5**
3.1 Sample chuck using drill press and hand tools 6

4 Sample masks **9**

5 NOT Gate for the Tachometer **10**

6 Tachometer **11**

7 Calibration **12**

8 Temperature on cycling of hV2 at 31% duty cycle (∼5000 RPM): With MOSFET-Low trace **12**

9 PBDB-T:PC71BM: additional detail on AFM for 3000 RPM, 60 s deposition **13**

10 Additional DIY health & safety **14**

11 Spin coating best practices **15**

12 Useful resources **15**

13 Existing spin coaters **15**
13.1 Open-source designs 15
13.2 Commercial spin coaters 16

14 After-market chucks **17**

1. Electronics for hV2

Figure 1 shows the circuit diagram for spin coater hardware version 2 (hV2). The circuit diagram for hV3 is included in the main manuscript. The primary development between these versions is of the input peripherals. hV2 uses potentiometers and buttons for operation while hV3 makes use of an incremental type rotary encoder.

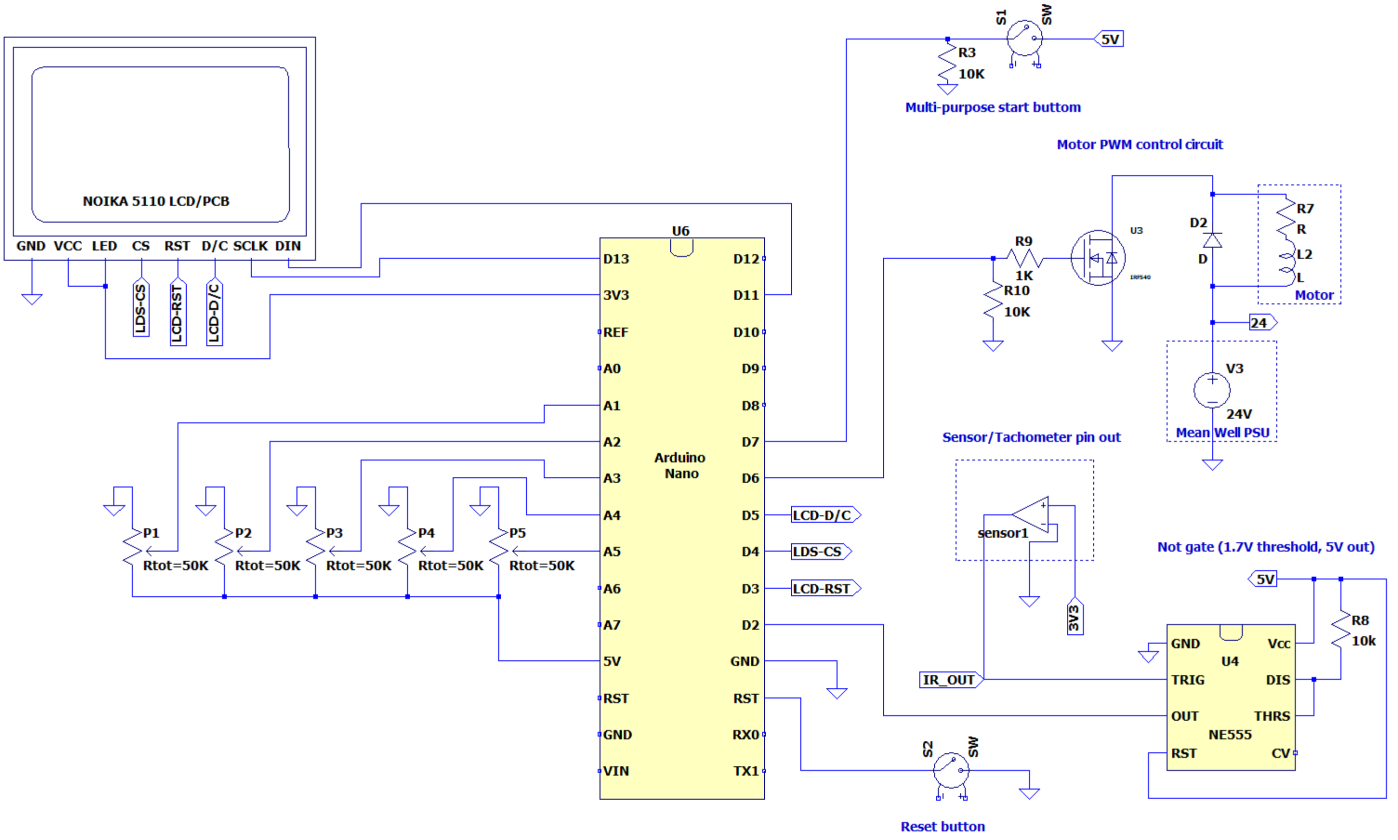

Figure 1: Circuit diagram for hardware Version 2

1.1 Assembled circuit

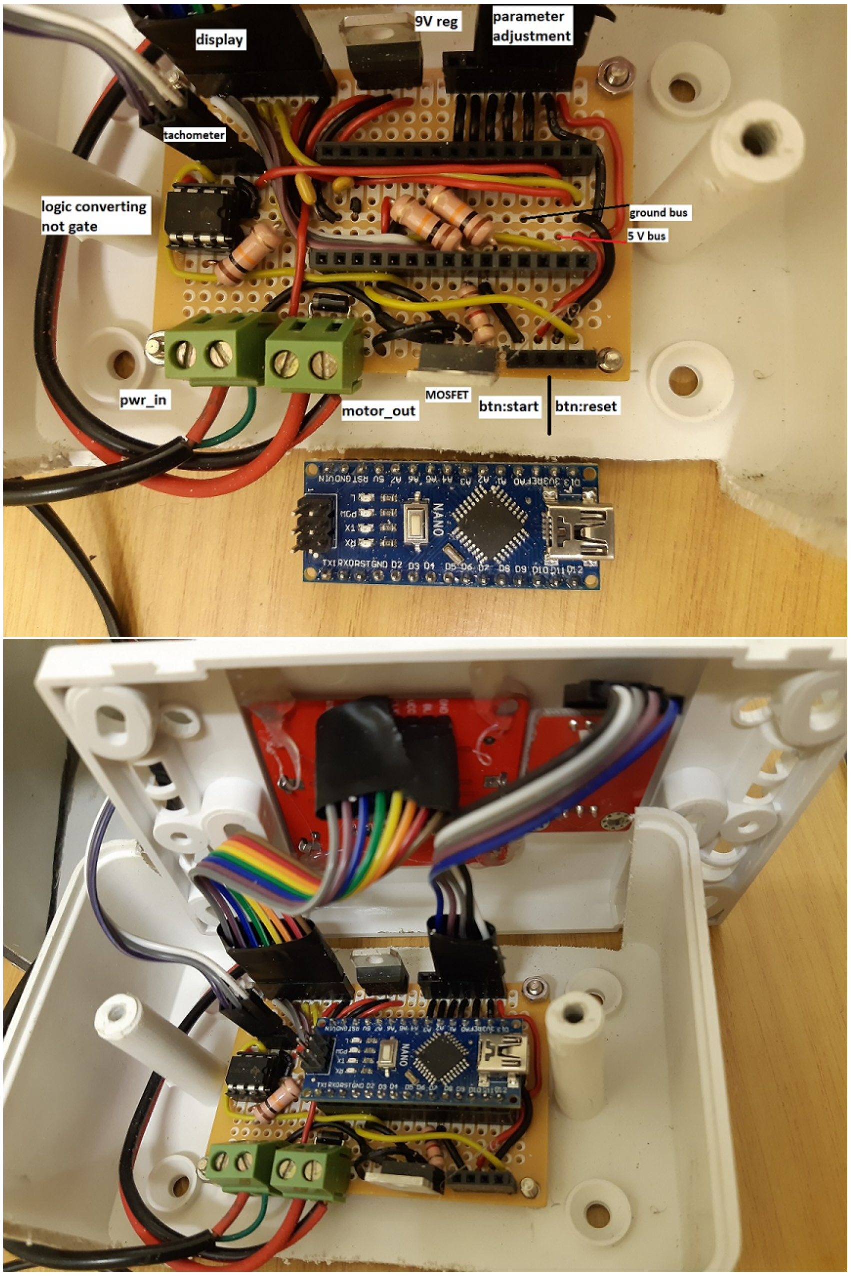

Figure 2: Electronics circuit board as assembled for hV3.

Figure 2 shows a labelled circuit board for hV3, including the unused "btn:start" and "btn:reset" header inherited from the design of hV2. The rotary encoder is connected to the "parameter adjustment" header. The mounting of the electronics board for hV2 is detailed in the hV2 assembly SI document.

For the parameter adjustment header, in hV2 the inputs consist of 5 potentiometers each connected to an analogue read pin (A2 to A6). In hV3 pins A0 to A2 are used for digital signals from the rotary encoder. The choice of pins is convenience based and requires consistency with the corresponding *pin* variables in the Arduino code. The power delivery in hV2 and hV3 differ, the Nano microcontroller in hV2 is powered through the USB cable by a 5.2 V step-down converter, while hV3 has an on-board 9V regulator as visible in Figure 2 and as described in the main manuscript.

2. Software flow diagram for hardware versions 2 and 3 (software versions 1.4 and 2.3)

The Arduino code written for hV2 (software 1.4) and hV3 (software 2.3) differ primarily in the handling of inputs used to program the run parameters as shown diagrammatically in Figure 3.

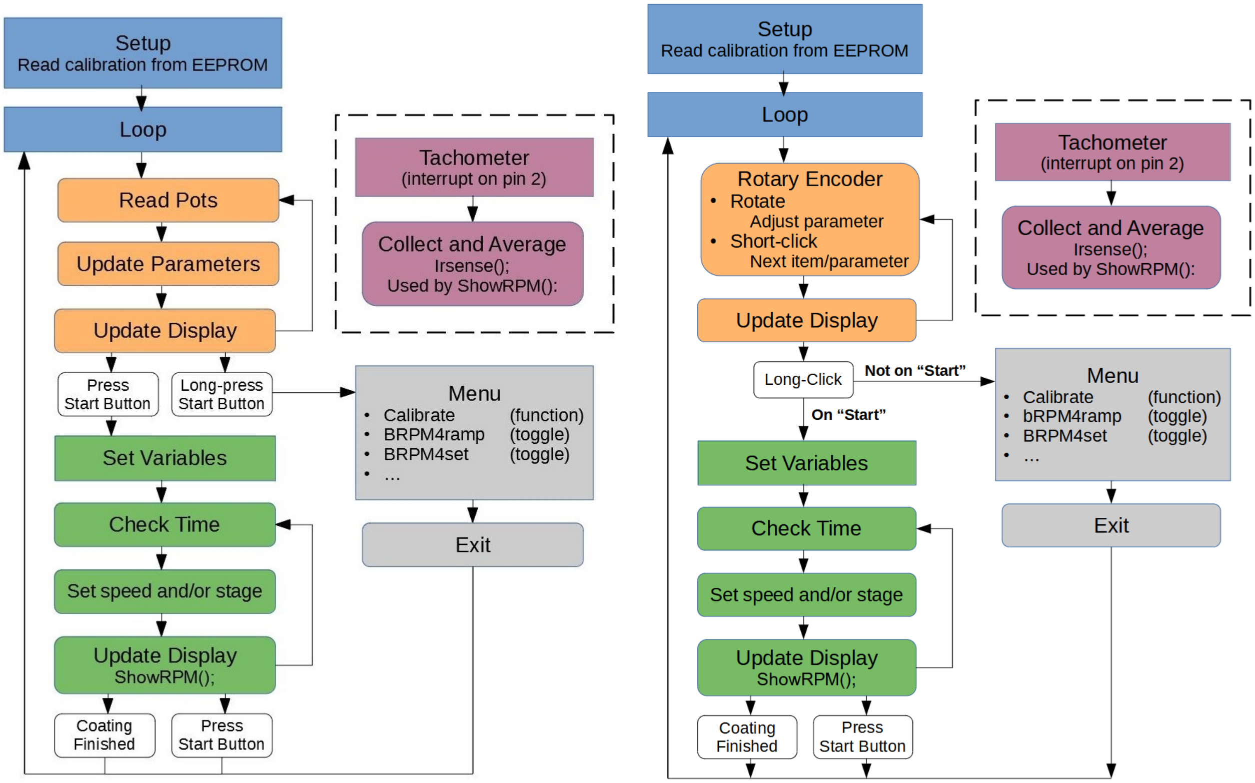

(a) Software version 1.4 (hV2)

(b) Software version 2.3 (hV3)

Figure 3: Flow diagrams of software versions 1.4 and 2.3 operation as used in hV2 and hV3 respectively.

3. Variations on sample chuck design

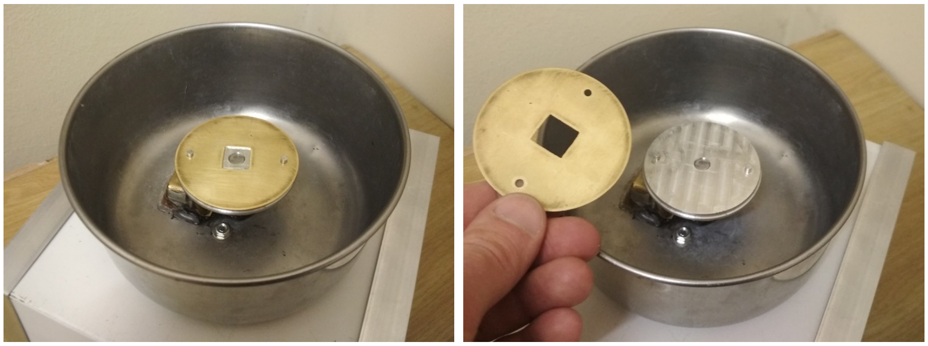

(a) (b)

Figure 4: Final sample chuck design used for hV2 employed smooth pins machined out of the same block of aluminium as the chuck itself. (a) Chuck with the sample mask and a glass substrate in place, (b) Chuck with the sample mask removed.

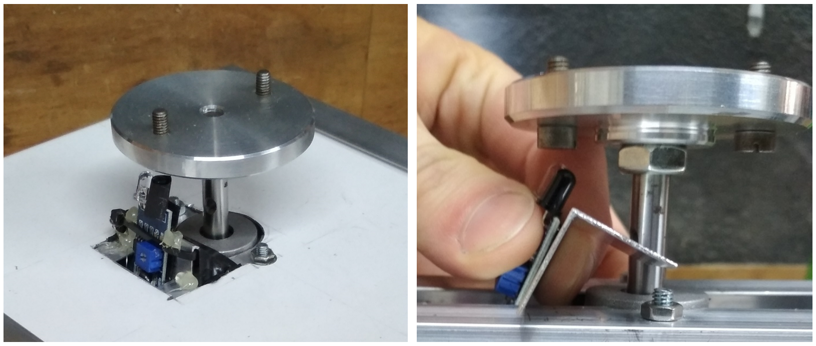

Figure 5: An earlier prototype of the sample chuck; threads were tapped into the aluminium chuck which held threaded bolts. These bolts acted as pins to hold the brass masks. To complete such a design the thread above the chuck surface needed to be machined off the bolts for easy placement and removal of the sample masks.

3.1 Sample chuck using drill press and hand tools

A more accessible alternative to a machined aluminium chuck was also considered. A sample chuck was produced from recycled plastic from a plasterers float, bending wire, and a 3mm drill bit using a drill press, hole saw bit, mini rotary tool, utility knife blade and sandpaper as detailed below. A plastic plasterers float can be purchased new for approximately $ 2-3.

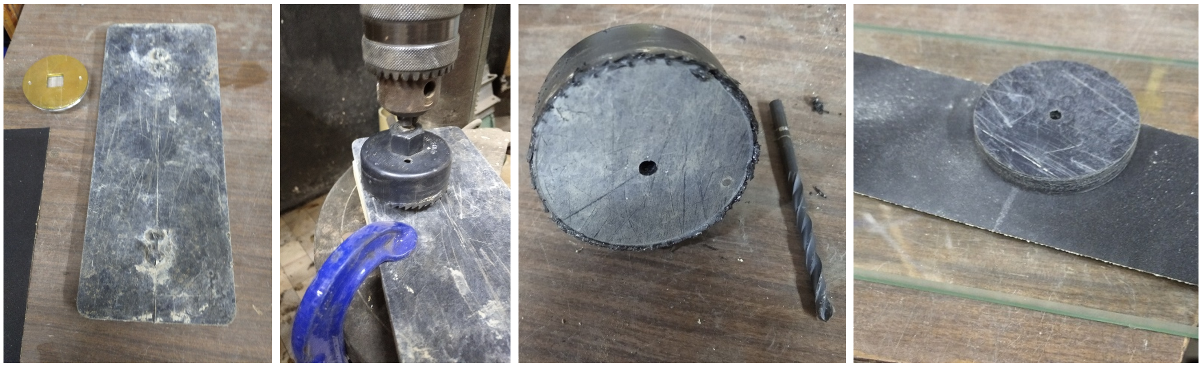

(a) (b) (c) (d)

Figure 6: Cutting a disk from a (broken) plasterers' float for a spin coater chuck.

- Figure 6b, The chuck was cut from the recycled plastic using a hole saw in a drill press. The plastic was tightly clamped to avoid it shifting during the cutting process.
- Figure 6c, For the case that the guiding bit of the hole saw arbour had a larger diameter than the shaft of the intended motor, a hole the intended size was first drilled with the drill press. After this, the guiding bit of the hole saw arbour was removed without shifting the drill press or plastic. The hole saw was then mounted in the drill press without its guiding drill bit (Figure 6b), and the plastic was slowly cut using the drill press.
  - For threaded motor shafts, an appropriate pilot hole is drilled. Thereafter a thread is tapped with a *tap* corresponding to the thread on the motor shaft.
- Figure 6d, Once cut the plastic disk was cleaned up with sandpaper. The sandpaper was placed on a sheet of glass which provides a reliably flat and smooth surface. [1] The plastic disk was rotated a few degrees at a time as it was worked across the sandpaper.

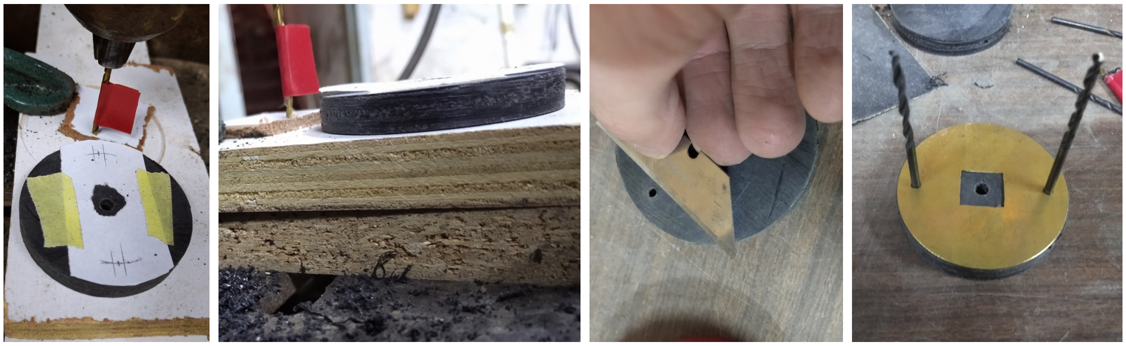

(a) (b) (c) (d)

Figure 7: Adding pins to the spin coater chuck to support the sample masks.

- Figure 7a, A paper template was prepared to mark the placement of the pins for the sample mask, the centre hole was 5 mm, and the pin spacing 45 mm centre to centre. Based on this, a vernier caliper was set to 20 mm and the pin positions were marked relative to the edge of the motor shaft hole.
- Figure 7b, The drill press was set up with a 3 mm bit and the intended depth was marked on the drill bit using red electrical tape.
- Figure 7c, These holes were "deburred" using a sharp blade.

[1] Glass does have a degree of flexibility as such is requires support. In the glass manufacturer links referenced, their float glass 4 to 6 mm has a manufacturing thickness tolerance of ± 0.2 mm. [supplier specification, archived-link (accessed 2022Jun25)]

- Figure 7d, The sample mask fit was tested by placing 2 x 3 mm drill bits in these holes.

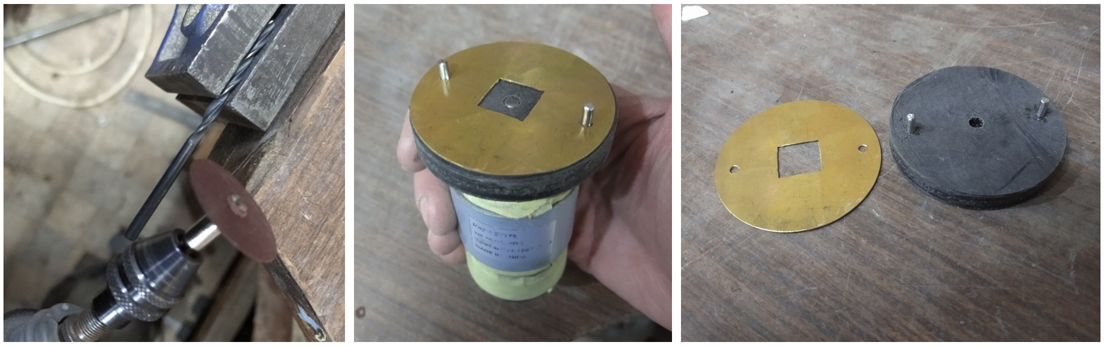

(a) (b) (c)

Figure 8: Adding pins to the spin coater chuck to support the sample masks.

- Figure 8a, The smooth shaft section of a 3 mm drill bit was cut into 2 pins using a mini grinder tool.
  - Drill bits offer a widely available source of steel pins in specific diameters.
- Figure 8b, The portion of these that was exposed when fitted into their holes was lightly ground down with the mini rotary tool grinding disk.
  - This increased the clearance between the pins and the sample mask for a looser fit. An alternative to this would be to use a slightly larger drill bit for the mask holes than used for the pin (i.e. 3.0 mm hole and 2.8 mm pins).

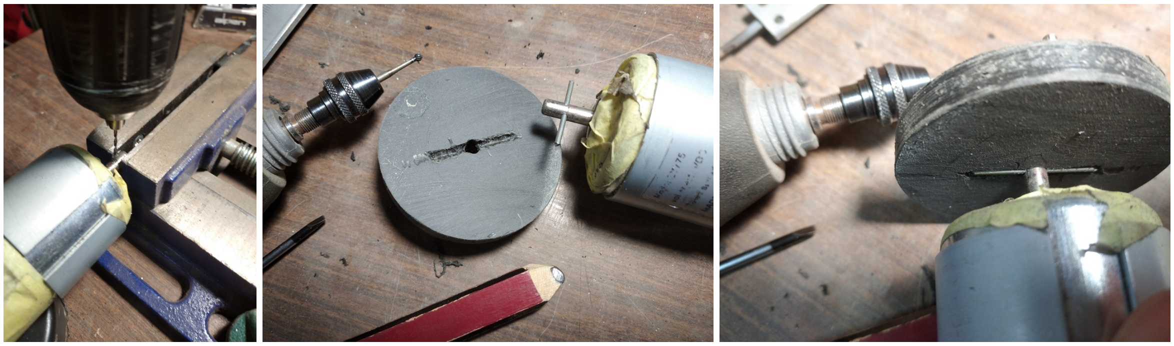

(a) (b) (c)

Figure 9: Making a seating, rotation locking mechanism for the chuck.

While the chuck fitted tightly on the motor shaft, for additional security a rotation locking mechanism was prepared as shown in Figure 9.

- Figure 9a, A 2 mm hole was drilled through the motor shaft, this should be performed at low speed with a drill press.
  - The low speed is suggested to reduce heat generation and prevent work hardening of the steel.
  - A drill press is a necessity to avoid a skew hole.
- Figure 9b, A bar shape was marked out on the chuck and a channel was cut into the chuck using a round bur in a mini rotary tool.
- Figure 9b, Bending wire ($\phi \approx 2$ mm) with a length corresponding to the length of the channel cut previously was placed though the motor shaft.

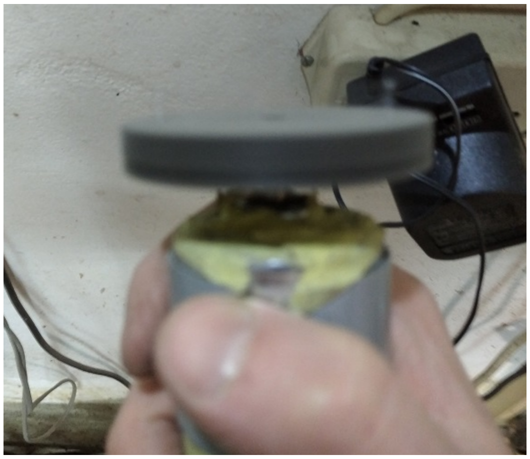

Figure 10: The spin coater chuck with rotation lock and sample mask pins rotating on a motor (photo).

The finished chuck was placed on the motor and a photo was taken during motor operation. The photo of the rotating chuck in Figure 10 shows very little precession, which would be due to miss-alignment of the chuck w.r.t the motor shaft. A large precession would be evident as a blur or transparency effect in the photo. This is by no means a quantitative test yet it shows that a reasonably useful spin coater chuck can be made with a drill press and hand tools.

A slight degree of miss-alignment between that chuck and motor shaft which was primarily attributed to the precession in the drill press and its stage being less than perfectly perpendicular to the drill bit *(The drill press in use was a repaired machine from 1986)*.

4. Sample masks

The sample masks were cut from 0.7 mm brass sheet using a CNC router (Figure 11). The brass sheet was fastened onto a wooden board for support during machining. The 3 mm holes were drilled followed by the centre opening, and lastly the perimeter was machined. The CNC router bits could only produce rounded corners for the centre hole, these inner corners were squared with a triangular jewellers file.

Similar brass work has been done by the author using a standard drill to make holes, followed by a jewellers saw to cut the centre shape and the perimeter.

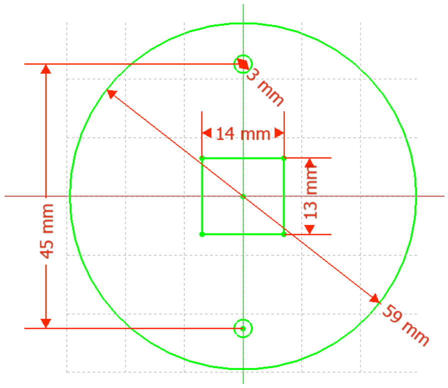

(a) A sample mask for hand-cut 12.5 x 12.5 mm samples.

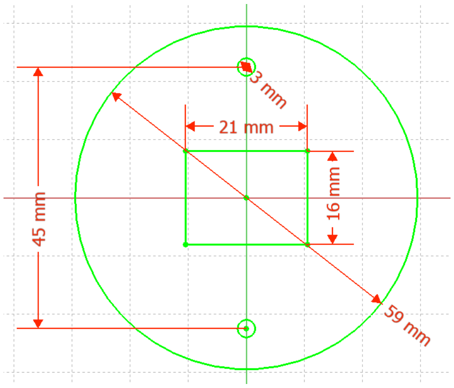

(b) A sample mask for 20 x 15 mm samples.

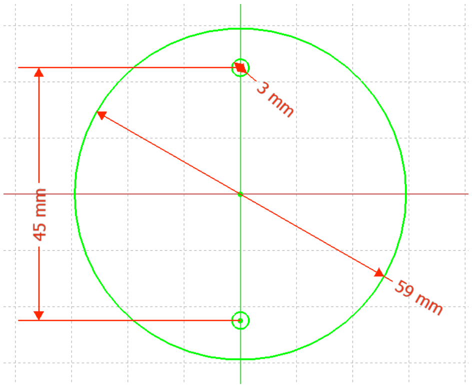

(c) A blank sample mask for later machining to accommodate an unaccounted for sample size.

Figure 11: Sample masks measurements, the sizes of the openings are larger than the intended samples for accommodation of any inaccuracies when using hand cut samples.

5. NOT Gate for the Tachometer

The NOT gate used alongside the tachometer originates from the availability of NE555 chips at the time and their ability to respond to relatively low ”trigger” voltages with short response times. Figure 12 shows the simulated response of the NOT gate on a millisecond timescale. The ”trigger” threshold voltage of the NOT gate is 1.67 V with the circuit using at most 2.1 mA during its operation. The LM1117 voltage regulator of the Arduino Nano compatible microcontroller has a maximum current draw specification of 800 mA making this a marginal power consumption compared to the supply capacity. These properties were found to be well suited to the application. An alternative is a MOSFET based logic level converter which consists of a MOSFET and two resistors.

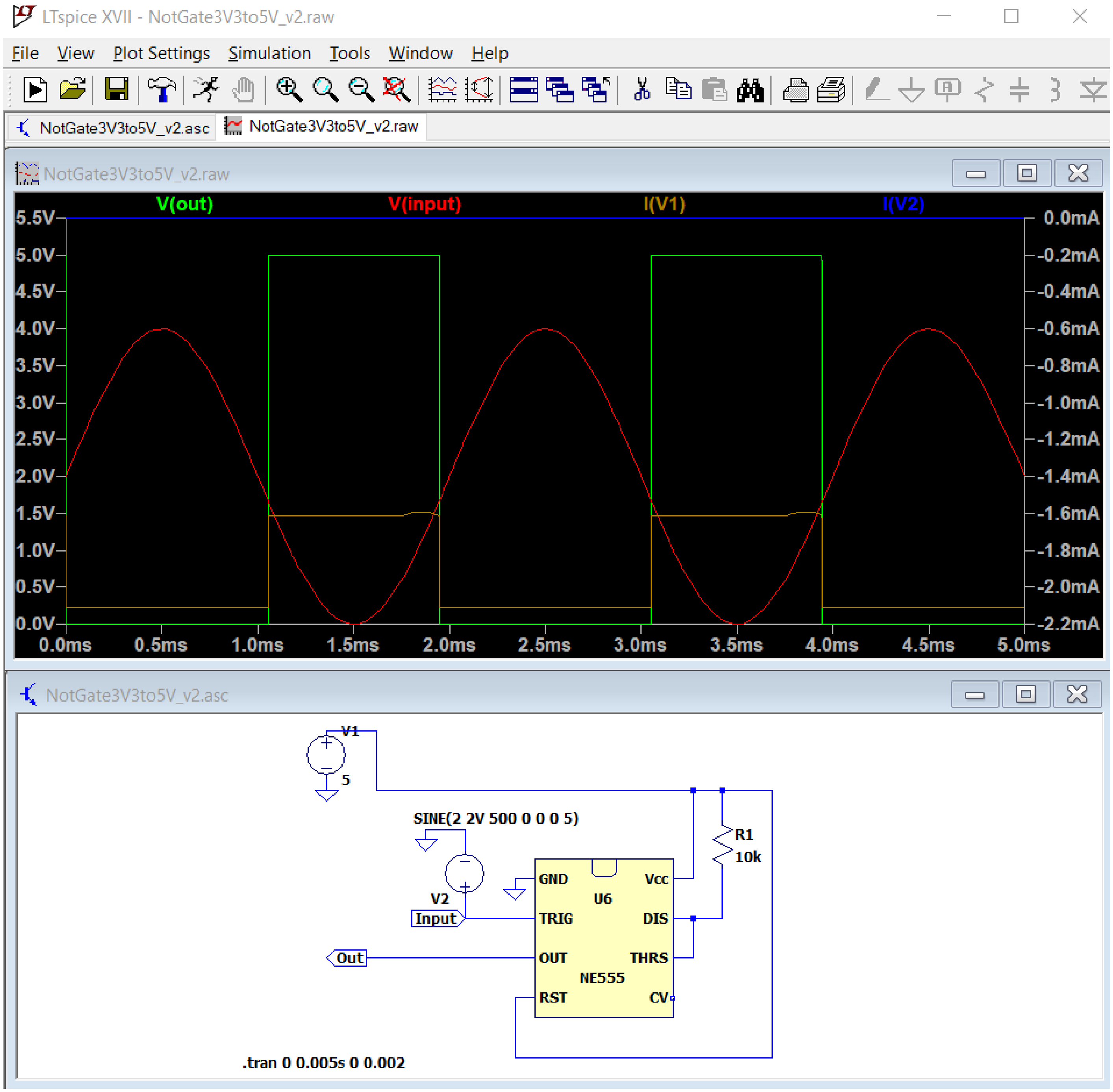

Figure 12: LTspice simulation of the NE555 based NOT gate with an input sine wave of 500 Hz (30000 cycles/min).

6. Tachometer

The tachometer was based around an “Infrared Obstacle Avoidance Sensor” [link, archived-link] as per Figure 13. hV2 used an infra-red sensor, the light emitted by this sensor is reflected off aluminium or is diffused and absorbed by sandpaper on the underside of the chuck. hV3 uses a brass reflector attached to the motor shaft on its rear end. The brass reflector was painted black on the inner-side and covered with aluminium tape on the outer-side for reflectivity contrast.

The conditions of a signal to be read as a ”guaranteed” digital high requires a voltage between $0.6V_{CC}$ and $V_{CC} + 0.5V$ for the ATmega328P chip in the Nano, where V_{CC} is the ATmega328P’s supply voltage of 5.0 V for this system. This translates to a signal between 3.0 and 5.5 V. For a digital low this value is between $-0.5V$ and $0.3V_{CC}$ $(1.5V)$. [ATmega328P_Datasheet, archived-link]

The tachometer was powered with 3.3 V and the output signal was measured as 3.2 V. Considering the digital logic level voltage windows of an ATmega32P microcontroller this should have been sufficient for reliable operation, but was found to not be the case during early experimentation. The use of NE555 chip to make a logic converting NOT gate as per Figure 12 improved reliability of the tachometer reading.

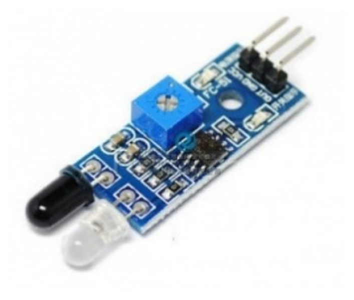

(a) Infra-red (IR) proximity sensor used in both hV2 and hV3.

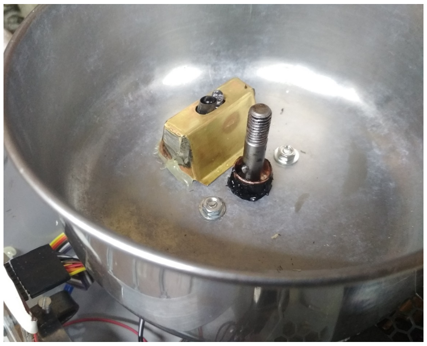

(b) IR proximity sensor in hV2.

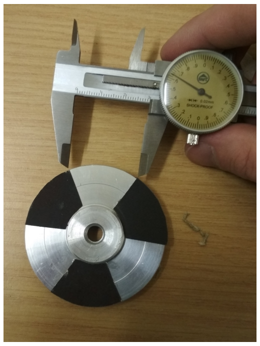

(c) Absorber/reflector pattern under the chuck in hV2.

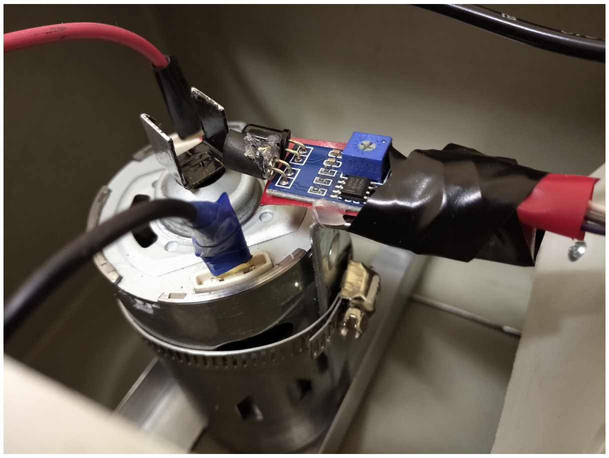

(d) IR sensor and reflector in hV3.

Figure 13: Tachometer sensor and its placement

7. Calibration

The calibration data for hV2 was fitted with a 6th order polynomial as shown in Figure 14, $R^2 = 0.9997$. The spin coater uses the raw calibration data for its operation while the calibration was fitted for the purpose of calculating a residual standard error, σ. σ was calculated to a value of 31.3 RPM as used in the *Validation and characterization* section of the main paper, using $\sigma = \sqrt{\frac{\sum(X_{Raw}-X_{Fitted})^2}{n-2}}$ where X refers to the RPM values and n is the number of collected data points.

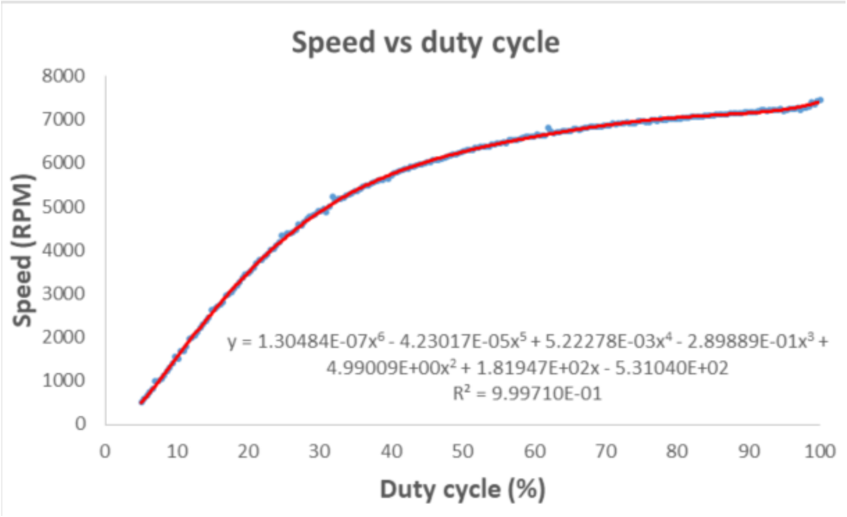

Figure 14: The relationship between motor speed and duty cycle at room temperature for the 24V DC motor controlled through a n-channel MOSFET with an Arduino Nano compatible microcontroller as used in hV2. (blue) Collected data, (Red) Fitted 6th order polynomial.

8. Temperature on cycling of hV2 at 31% duty cycle (~5000 RPM): With MOSFET-Low trace

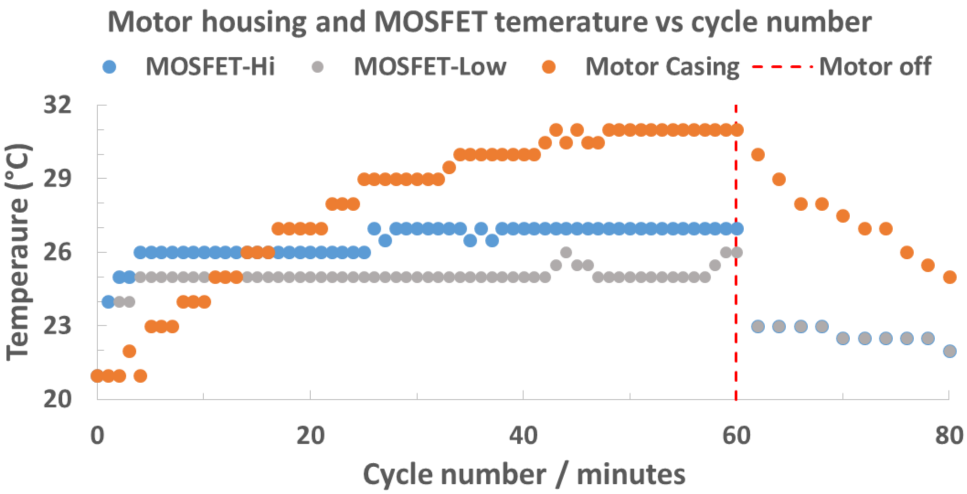

Figure 15: Motor housing and MOSFET temperature as a function of cycle number with the additional MOSFET-Low trace.

The MOSFET-Low trace in Figure 15 rapidly reaches 25 ° C. At cycles 44, 59 and 60 the temperature reaches 26 ° with nearby values of 25.5 ° C representing instability between a reading of 25 and 26 during the cycle ° C. This is with consideration of a temperature measurement resolution of 1 ° C.

9. PBDB-T:PC71BM: additional detail on AFM for 3000 RPM, 60 s deposition

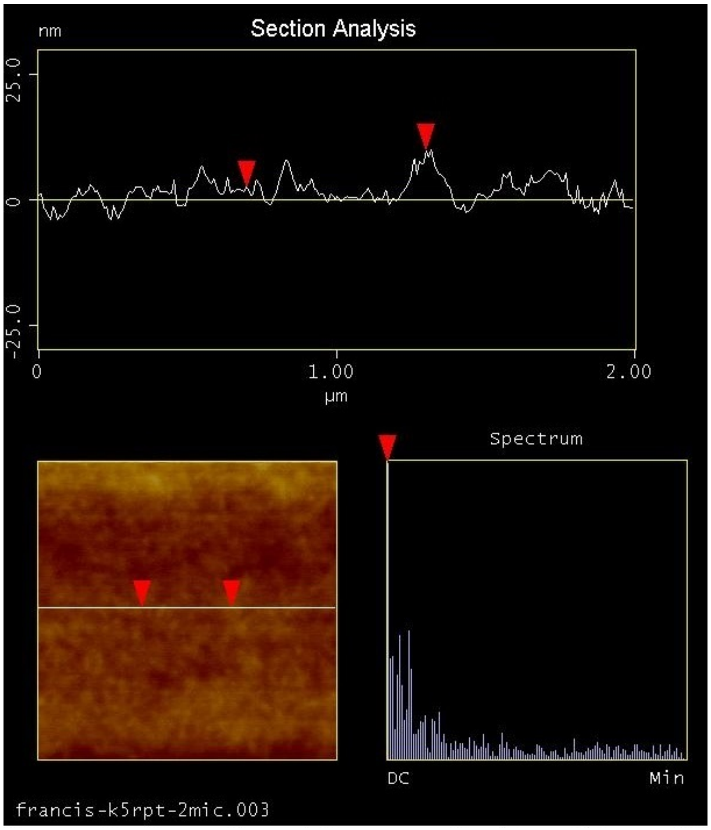

Parameter	Value
L	601.56 nm
RMS	2.515 nm
lc	DC
Ra(lc)	1.910 nm
Rmax	10.887 nm
Rz	5.739 nm
Rz Cnt	valid
Radius	2.473 μm
Sigma	5.039 nm

Figure 16: Tapping mode AFM of PBDB-T:PC71BM (1:1) thin films, spin coated for 60 s at 3000 rpm showing a 3 μm line trace.

Figure 16 shows a AFM line trace in the top left corner with parameters from this trace on the right. Here we see a difference between the maximum and minimum points of *Rmax*, 10.887 nm $\approx$ 11 nm.

10. Additional DIY health & safety

The following precautions provide **some** of the most important relevant hazards and precautions. Some points are derived from sources including [1].

Electrical hazards

- Check the wiring on all tool and equipment for damage or bad insulation. Do not use equipment with hazardous wiring.
- Do not use damages tools and equipments.
- Do not work with electricity in wet areas.

Safety practices

- For any work with or around potentially dangerous equipment, minimise the potential dangers, ensure a clean and clear workspace and ensure you have stable footing, especially when working with power tools such as angle grinders.
- Any materials being cut or worked on should be well secured to a sturdy surface.
- Work with a colleague present in the area.
- Remove jewellery and metal objects when working with motorised tools or electricity.

Personal Protective Equipment (PPE)

- Wear safety glasses.
- Don't wear loose clothing.
- Secure long hair.
- Wear protective footwear.
- Use hearing protection where appropriate such as for angle grinders.

Chemical Safety

- Read the material safety data sheets (MSDS) for all chemicals used during spin coating and follow the appropriate safety precautions.
- Operate the spin coater in a well ventilated area, glove-box or fume-hood as appropriate for the chemicals in use.
- Wear appropriate PPE including gloves, safety glasses, lab coat, etc.

Rotary motor safety

- Keep the sample chamber clear of obstructions.
- Do not touch the chuck or any moving parts while they are in motion.
- Wear appropriate PPE including gloves, safety glasses, lab coat, etc.
- The spin coater should not be operated in the presence of loose hair, loose clothing, loose jewellery or anything that could get caught in and/or wrapped around moving parts.

11. Spin coating best practices

- When using **vacuum chucks** on commercial spin coaters. the precursor solution can leave behind residues, and any such precursor solution drawn by the vacuum chuck has the potential to gradually build up a residue *gluing* the chuck to the motor shaft. As such it was found best to remove and clean the chuck after every use. This was especially critical for solutes such as lead halides in dimethylformamide where the low solubility of the lead salt readily causes residue deposits that require harsh solvents to remove.
- In glove boxes or other closed off environments where research involving multiple chemistries take place, contamination from the spin coating process can be deleterious as such these environments should be closely monitored when introducing spin coating for potential impacts on adjacent works.
- The precursor solutions can be passed through a sub micrometer syringe filter to remove undissolved particles or contaminants from the solution which could be the source of "comet" film defects. [2]
- When applying precursor solution, a micropipette allows the application of a reliable, reproducible quantity which can be finely tuned to reduce wasting of precursor solution. An example of an open-source micropipette build can be found at [3]

12. Useful resources

- *Ossila's spin coating guide,*
  Ossila provides a spin coating guide which covers wide range of concepts and considerations including spin coating theory, practical considerations and handling defects. Among this information they mention vacuum warping of thin substrates and the additional maintenance required with vacuum chucks as the vacuum often draws in the precursor ink/solution. [Ossila spin-coating, archived-link (accessed 8 Nov 2021)]
- *Spin coating: art and science,*
  Birnie lays out the dominant physics in spin-coating, coating defects and the causes of in the chapter "Spin coating: art and science" of the book "Chemical Solution Deposition of Functional Oxide Thin Films". This includes the work of Emslie, Bonner and Peck where *flow* was described as a primary to the evolution of film thickness, determined by viscous forces, density and rotational speed. Meyerhofer expanded this work by including evaporation effects into the equations which become dominant after the rate of flow dominated change in thickness declines. In the section where defects are discussed, these include defects related to solvent evaporation, surface tension, viscosity and contaminants. The causes of these defects are discussed with direct or indirect implementations of how to address them. [2, 4, 5].

13. Existing spin coaters

13.1 Open-source designs

- Sadegh-cheri's spin coater [6]
  Double sided tape sample mounting
  1000-9000 rpm
  Brushless DC motor from a computer hard
  https://pubs.acs.org/doi/full/10.1021/acs.jchemed.9b00013 (accessed 2021Nov08)

- Bianchi *et al.* [7]
  Spin coater based on brushless dc motor of hard disk drivers
  Vacuum sample mount
  0-10000 rpm
  Brushless DC motor from a computer hard

- Yetkin Akyüz Electrical Electronics Engineer - Düzce University [8]
  Gravity chuck, 4 steel flat bar sections with a slide channel in which a screw fixed the slides to the chuck. This allows sample size adjustment using a screwdriver with the minimum size defined by the width of

the flat bar.
Programmable up to 10 stages
24 V 9000 rpm DC motor [project page link, archived-link (accessed 2021Nov08)]

- Segura *et al.* Mechanical and Electronic Systems of an Open Source Based Spin and Dip Coater [9]
  Double sided tape sample mounting
  300 - 10000 rpm speed control by Arduino Uno hardware PWM signal (byte value of 0-255)
  24 V, 12 W DC motor
  https://revistapolitecnica.epn.edu.ec/ojs2/index.php/revista_politecnica2/article/view/561 (accessed 8Nov2021)

- Ben Krasnow photoresist spin coater [10]
  Mounting set for microscope slides, flat chuck with 5 flat-head screws, 4 fixed and one removed and replaced for sample mounting.
  Quad-copter motor driven through an ESC controller. These types of motors and controller combinations can offer control over the motor acceleration and deceleration rate as well as speed readings either by monitoring the motor windings during rotation or through the hall sensor in variants which have one included. Quad-copter motors frequently run at a few 10 000's rpm. As such care needs to be taken to ensure the motor can go slow enough. A speed rage from a few hundred to a few thousand rpm is often required for a spin coater.
  https://benkrasnow.blogspot.com/2016/02/design-and-build-spin-coater.html (accessed 8Nov2021)

13.2 Commercial spin coaters

**Supplier**	**Model**	**Chuck type**	**Speed RPM**	**Price**	**Additional**
SETCAS [11]	SETCAS KW-4A	Vacuum	500 - 8500	$ 2000 (Nov2021) Cheapest of their range	• Substrate size ϕ 5- 100 mm<br>• Two-stage<br>• Includes Vacuum pump<br>• Includes 3 chucks<br>• Motor speed stability ± 1%, Tachometer ± 10 rpm<br>• 2 Year warrantee
	https://www.setcas.online/product-page/kw-4a (accessed 8Nov2021)				
SPI [12]	SPI KW-4A 220V	Vacuum	500 - 8000	$ 5505.79 (Nov2021)	• Two-stage<br>• Substrate size up to 152 mm (6")<br>• Speed stability ¡ 1%<br>• (A diaphragm pump is recommended for operation)
	https://www.2spi.com/item/12170-ax/spin-coater/ (accessed 8Nov2021)				
MTI Corporation [13]	Compact Spin Coater VTC-100A 220V	Vacuum	500 - 8000	$ 3988.00 + $ 598.20 not in USA warrantee surcharge (Nov2021)	• Two-stage<br>• 1,2 and 4" vacuum chucks included<br>• 24 V DC motor rated 150 W Max<br>• 1 Year warranty with lifetime support
	https://www.mtixtl.com/ProgrammableCompact SpinCoater0-8000rpm4waferMax-VTC-100A.aspx (accessed 8Nov2021)				
Ossila [14]	Ossila Spin Coater	Gravity	120 - 6000	£1900 (Nov2021)	• Digitally stored step profiles up to 50 steps each<br>• Built in spirit level<br>• Includes a chuck<br>• 2 year warrantee
	https://www.ossila.com/products/spin-coater (accessed 8Nov2021)<br>https://web.archive.org/web/20210428130823/https://www.ossila.com/products/spin-coater				

14. After-market chucks

- Vacuum chucks for SETCAS KW series starting at $ 100 (21 Oct2021, archived-link). Their website reported the starting price as $ 130 in Nov2021 and $ 300 in Aug 2022 archived-link, it is suspected the difference was related to stock availability of their lower cost options.
  https://www.setcas.online/product-page/vacuum-chuck (accessed 8Nov2021)

- Vacuum chucks for SPI KW-4A
  From SPI starting at $91.80 for aluminium and $153.00 for PTFE (Nov2021)
  https://www.2spi.com/item/z12173/
  https://www.2spi.com/item/z12173t/

- Anti-corrosive vacuum chucks with O-Rings of different sizes FOR VTC-100PA - MTI-VTC100PA
  Price not specified but adding an extra O-Ring for the ϕ 10 mm chuck is marked as $ 25.95, it is unspecified what advanced material this $ 25.95 O-Ring is made from.
  https://www.mtixtl.com/VacuumchuckswithdifferentsizesFORVTC-100PAMTI-VTC100PA.aspx (accessed 8Nov2021)

- Ossila spin coater chucks
  £199 for standard or £249 for a custom chuck Nov2021. Ossila also provides *.STL* and *.SLDPRT* files to allow their customers to produce their own chucks.
  https://www.ossila.com/products/spin-coater-chucks (accessed 8Nov2021)

References

[1] J. Ojeme, E. Raymond, Occupational safety and health practices required by electrical/electronics technology graduates in north central nigeria (2021).

[2] D. P. Birnie, Spin coating: art and science, in: Chemical Solution Deposition of Functional Oxide Thin Films, Springer, 2013, pp. 263–274.

[3] M. D. Brennan, F. F. Bokhari, D. T. Eddington, Open design 3d-printable adjustable micropipette that meets the iso standard for accuracy, Micromachines 9 (4) (2018) 191.

[4] A. G. Emslie, F. T. Bonner, L. G. Peck, Flow of a viscous liquid on a rotating disk, Journal of Applied Physics 29 (5) (1958) 858–862.

[5] D. Meyerhofer, Characteristics of resist films produced by spinning, Journal of Applied Physics 49 (7) (1978) 3993–3997.

[6] M. Sadegh-cheri, Design, fabrication, and optical characterization of a low-cost and open-source spin coater, J. Chem. Educ. 96 (6) (2019) 1268–1272. doi:https://doi.org/10.1021/acs.jchemed.9b00013.

[7] R. F. Bianchi, M. Panssiera, J. Lima, L. Yagura, A. Andrade, R. M. Faria, Spin coater based on brushless dc motor of hard disk drivers, Prog. Org. Coat. 57 (1) (2006) 33–36. doi:https://doi.org/10.1016/j.porgcoat.2006.05.004.

[8] Y. Akyüz, Design and fabrication of a cost-effective spin coater, (accessed: 2021-11-08) (2020).
URL https://github.com/yetkinakyz/SpinCoater

[9] R. Dabirian, V. H. Guerrero, D. C. L. Matovelle, L. J. Segura, Mechanical and electronic systems of an open source based spin and dip coater, Revista Politécnica 37 (2) (2016) 53–53.

[10] Y. Akyüz, Design and build a spin coater, (accessed: 2021-11-08) (2016).
URL https://benkrasnow.blogspot.com/2016/02/design-and-build-spin-coater.html

[11] SETCAS LLC, Setcas llc — kw-4a spin coater, (accessed: 2021-11-08).
URL https://www.setcas.online/product-page/kw-4a

[12] SPI KW-4A, Setcas llc — kw-4a spin coater, (accessed: 2021-11-08).
URL https://www.setcas.online/product-page/kw-4a

[13] MTI Corporation, Compact spin coater - vtc-100a, (accessed: 2021-11-08).
URL https://www.mtixtl.com/ProgrammableCompactSpinCoater0-8000rpm4waferMax-VTC-100A.aspx

[14] Ossila Limited, Spin coater — low price compact spin coating system, (accessed: 2020-02-04).
URL https://www.ossila.com/products/spin-coater?variant=1200242585
